# Turning up a new pattern: Identification of cancer-associated fibroblast-related clusters in TNBC

**DOI:** 10.3389/fimmu.2022.1022147

**Published:** 2022-10-06

**Authors:** Jindong Xie, Shaoquan Zheng, Yutian Zou, Yuhui Tang, Wenwen Tian, Chau-Wei Wong, Song Wu, Xueqi Ou, Wanzhen Zhao, Manbo Cai, Xiaoming Xie

**Affiliations:** ^1^ Department of Breast Oncology, Sun Yat-sen University Cancer Center, State Key Laboratory of Oncology in South China, Collaborative Innovation Center for Cancer Medicine, Guangzhou, China; ^2^ Breast Disease Center, The First Affiliated Hospital, Sun Yat-Sen University, Guangzhou, China; ^3^ Department of Radiotherapy, The First Affiliated Hospital, Hengyang Medical School, University of South China, Hengyang, Hunan, China

**Keywords:** triple-negative breast cancer, cancer-associated fibroblasts, tumor microenvironment, machine learning, prognostic model

## Abstract

Growing evidence indicates a connection between cancer-associated fibroblasts (CAFs) and tumor microenvironment (TME) remodeling and tumor progression. Nevertheless, how patterns of CAFs impact TME and immunotherapy responsiveness in triple-negative breast cancer (TNBC) remains unclear. Here, we systematically investigate the relationship between TNBC progression and patterns of CAFs. By using unsupervised clustering methods in the Molecular Taxonomy of Breast Cancer International Consortium (METABRIC) dataset, we identified two distinct CAF-associated clusters that were related to clinical features, characteristics of TME, and prognosis of patients. Then, we established a CAF-related prognosis index (CPI) by the least absolute shrinkage and selection operator (LASSO)-Cox regression method. CPI showed prognostic accuracy in both training and validation cohorts (METABRIC, GSE96058, and GSE21653). Consequently, we constructed a nomogram with great predictive performance. Moreover, the CPI was verified to be correlated with the responsiveness of immunotherapy in three independent cohorts (GSE91061, GSE165252, and GSE173839). Taken together, the CPI might help us improve our recognition of the TME of TNBC, predict the prognosis of TNBC patients, and offer more immunotherapy strategies in the future.

## Introduction

Breast cancer remains the primary disease burden in women worldwide, according to GLOBOCAN 2020 estimates (1). Triple-negative breast cancer (TNBC) is the most invasive molecular subtype of breast cancer that lacks the expression of hormone receptors and human epidermal growth factor receptor 2 (HER2). Due to chemo-resistance and poor prognosis, the therapy of TNBC is challenging and considered as a “black hole” in contrast to other subtypes of breast cancers (2). Therefore, it is necessary to explore the mechanism of the development and progression of TNBC, and effective models are crucial to fill up the gap of TNBC treatment.

It has been proven that the tumor microenvironment (TME) is closely linked to tumor development and progression (3, 4). Stromal cells and immune cells make up the majority of cells in TME. Growing evidence indicates that stromal cells contribute to tumor progression (5, 6). Among these stromal cells, cancer-associated fibroblasts (CAFs) are the major component and play a vital role in tumor proliferation and invasion (7). Previous studies have demonstrated that CAFs can interact with tumor cells and regulate the metastasis of TNBC (8, 9). Clinical trials aimed at targeting CAFs have been conducted in recent decades; however, most of them are still in progress (7). Moreover, various CAF markers have been identified, but few of them make into clinical application because of the inherent heterogeneity (10). Most CAFs originate from adjacent normal tissues with the induction of oxidative stress, chemokines, and cytokines derived from tumor cells (11). Previous studies have found that CAFs could be divided into myofibroblastics (myCAF) and inflammatory (iCAF) subgroups (12). Furthermore, single-cell analysis reveals that, in breast cancer, myCAF can be divided into five different clusters (ecm-myCAF, TGFβ-myCAF, wound-myCAF, IFNαβ-myCAF, and acto-myCAF), and ecm/TGFβ-myCAF clusters are related to the resistance to immunotherapy (13). Immunotherapy has recently emerged as a hopeful strategy in breast cancer treatment (14). Hence, focusing on the role of CAFs in the TME might make a contribution to breast cancer treatment, especially immunotherapy.

How patterns of CAFs impact the characteristics of TME and the efficacy of immunotherapy in TNBC remains unclear. Here, we systematically conducted a study on CAFs, and established a novel indicator, CAF-related prognosis index (CPI), to predict the prognosis and responsiveness of immunotherapy. This study might help improve our recognition of TME of TNBC, predict prediction of prognosis of TNBC patients, and offer more reliable immunotherapy strategies in the future.

## Materials and methods

### Data sources

Patients who fulfilled the following selection criteria were considered: (a) diagnosed with histologically confirmed breast cancer; (b) molecular subtype with ER negative, PR negative, and HER2 negative; (c) available survival data; and (d) remove technical replications if necessary. The DNA microarray data of 298 TNBC patients were collected from the METABRIC dataset (Dataset ID: EGAS00001001753). Corresponding clinical features, copy number variation (CNV) information, and masked somatic mutation information were also downloaded. We acquired independent validation cohorts (ID: GSE96058 and GSE21653) and immunotherapy cohorts (ID: GSE91061, GSE165252, and GSE173839) from the Gene Expression Omnibus (GEO) database. The probes were mapped using the R package “AnnoProbe”. Expression data of the model genes in TNBC and normal tissues were downloaded from The Cancer Genome Atlas (TCGA). CAF-related genes were collected from previous studies and manual collation (15, 16).

### Multi-omics landscape of the CAF-related genes

The locations, expression levels, and relationships of the CAF-related genes were visualized by applying the “RCircos” R package (17). Protein–protein interaction (PPI) network analysis was performed and embellished with the help of STRING and Cytoscape (18, 19). Multi-omics information was synthetically shown using the “ComplexHeatmap” R package (20).

### Unsupervised clustering of the CAF-related genes

The expression of CAF-related genes was enrolled to accomplish consensus clustering (CC) to identify the CAF-related clusters in TNBC by using the “ConsensusClusterPlus” package (21). Principal component analysis (PCA) was performed by the R package “scatterplot3d”.

### Relationships between the clusters with prognosis and clinical features

We compared the relationships between the clusters, prognosis, and clinical features of TNBC patients. The differences in overall survival (OS) time were assessed *via* Kaplan–Meier (K-M) analysis using the “survival” and “survminer” R packages. Furthermore, age, tumor size, PAM50 subtype, tumor location, pathologic N, clinical stage, and pathologic grade were incorporated into clinical features.

### Correlations of the clusters with TME characteristics

The differences between the clusters in biological processes were found by applying gene set variation analysis (GSVA), with the gene set derived from the MSigDB database (c2.cp.kegg.v7.2) (22). The compositions of 22 immune cells of each TNBC patient were assessed by the CIBERSORT algorithm (23). The expression of immune checkpoints between the clusters was also analyzed. In addition, we applied the ESTIMATE algorithm to obtain the immune and stromal scores of each patient (24).

### Identification of differentially expressed genes and functional enrichment

Subsequently, we screened out DEGs with the criterion of *adjust. p* < 0.05 and *|log2 Fold Change|* > 0.5 (R package “limma”) (25). The R package “clusterProfiler” was used to perform functional enrichment analysis (26).

### Construction of the CPI

We utilized univariate Cox regression to find DEGs linked to TNBC OS. The cutoff criterion was 0.05. We randomly split all TNBC patients in the METABRIC cohort into a training cohort and a validation cohort with the proportion of 6:4. We applied the least absolute shrinkage and selection operator (LASSO)-Cox regression method to construct the optimal signature in the training cohort (R package “glmnet”) (27). Finally, the signature exported the CPI of each patient as follows:


$$CPI=\sum_{i=1}^{7}Coefi*Expi$$

Coefi denotes the risk coefficient and Expi refers to the expression of each gene. To make plots more intuitionistic, we used a linear transformation to adjust the CPI (28).


$$\text{Normalized}\;CPI=\frac{CPI\;-min\left(CPI\;\right)}{\max\left(CPI\;\right)-min\left(CPI\;\right)}$$

The alluvial diagram was shown by the “ggalluvial” R package. The “stats”, “survival”, and “survminer” package were used to investigate the correlation between OS time and CPI. We applied the “timeROC” R package to finish receiver operating characteristic (ROC) curve analysis (29).

### The establishment and assessment of the nomogram

Clinical features (age, tumor size, pathologic N, and grade) and CPI were set together, and the prognostic nomogram was established by the multivariable Cox and stepwise regression analyses (R package “regplot”). We used “caret” and “rmda” packages to make calibration plots and DCA to evaluate nomogram efficacy.

### Statistical analysis

All statistical analyses were presented *via* R 4.1.2. Wilcoxon test was used for comparison of different clusters and CPI groups. The Spearman method was applied for correlation analysis. Chi-square test and Fisher’s exact test were applied for comparison of clinical features.

## Results

**Figure 1:**
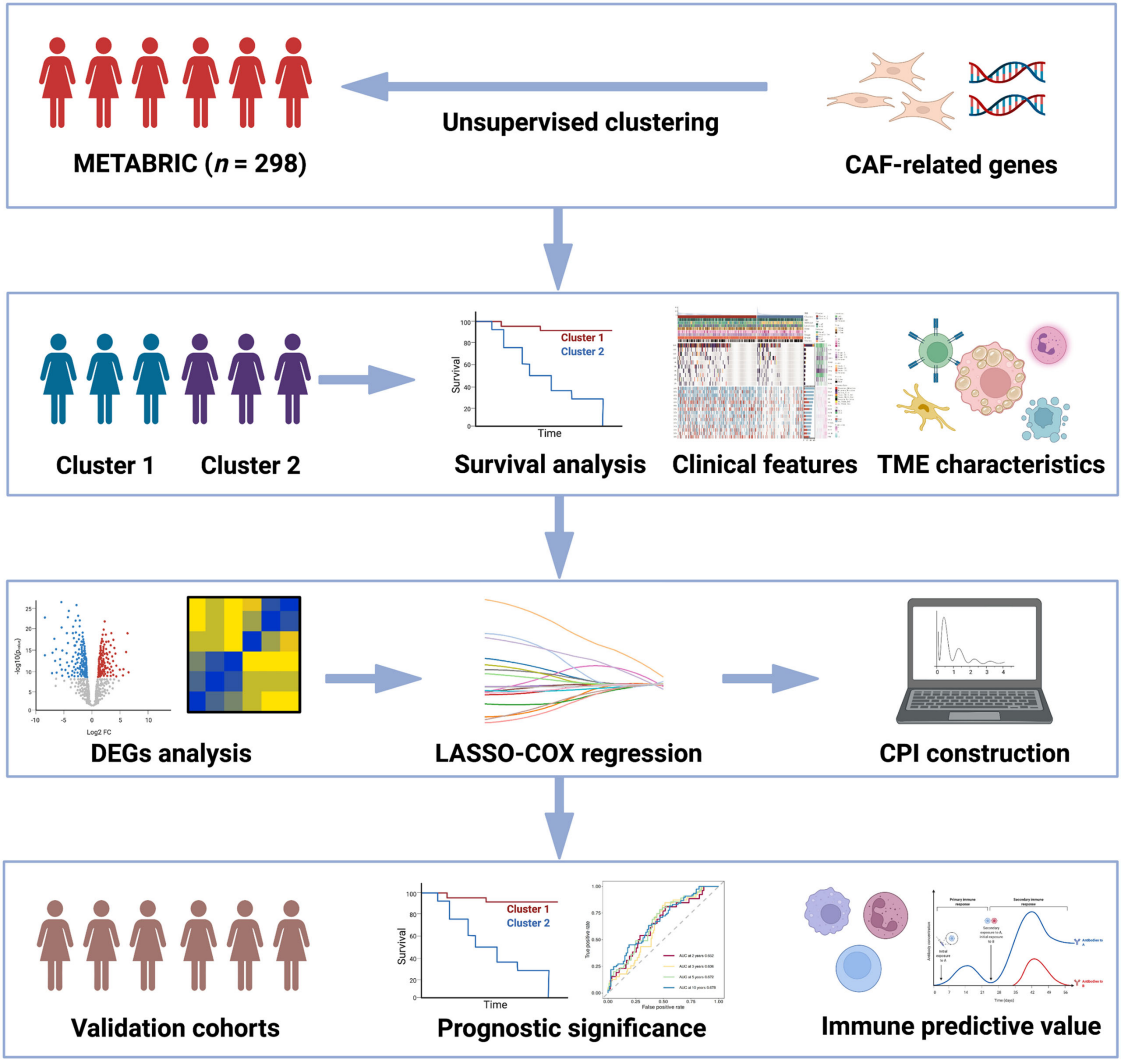
Flowchart for comprehensive analysis of the new CAF-related pattern in TNBC.

The workflow in our study is presented in **Figure 1**.

### General recognition of the CAF-related genes

**Figure 2:**
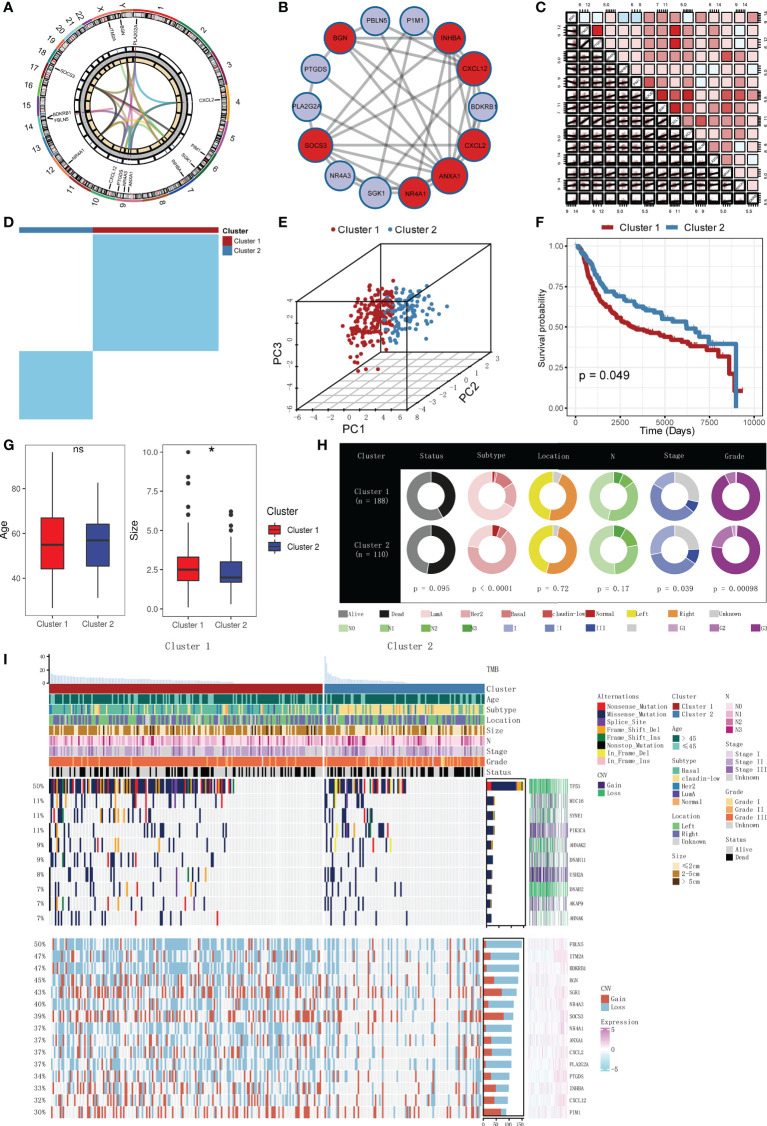
General recognition and unsupervised clustering of the CAF-related genes, and relationships between clusters with prognosis and clinical features. **(A)** Locations, expression levels, and links of the CAF-related genes on 23 chromosomes. **(B)** PPI network of the CAF-related genes. Different colors represent different clusters. **(C)** Correlations of each CAF-related gene (red: positive correlation; sky blue: negative correlation). **(D)** Unsupervised clustering based on the CAF-related genes in the METABRIC cohort (k = 2). **(E)** PCA analysis of two clusters (firebrick: cluster 1; steel blue: cluster 2). **(F)** K-M survival analysis of two clusters (firebrick: cluster 1; steel blue: cluster 2). **(G)** Boxplots of two clusters with age and tumor sizes (firebrick: cluster 1; steel blue: cluster 2; ns means no significance, and * means p < 0.05). **(H)** Doughnut diagrams of two clusters in survival status, PAM50 subtype, pathologic location, pathologic N, clinical stage, and pathologic grade. **(I)** Muti-omics landscape of samples in two clusters.

The locations, expression levels, and links of the CAF-related genes on their respective chromosomes are shown in **Figure 2A**. **Figure 2B** shows the PPI network among the CAF-related genes. Correlations of each CAF-related gene are exhibited in **Figure 2C**. We can see that most of the CAF-related genes were positively correlated.

### Unsupervised clustering of the CAF-related genes

To explore unidentified clusters of TNBC, CAF-related genes were employed to perform a CC analysis in the METABRIC cohort. We found that when *k* = 2, the differences among subgroups were the most obvious, which indicated that *k* = 2 might be optimum. Based on the selected *k* value, the cohort was divided into cluster 1 (*n* = 188) and cluster 2 (*n* = 110) (**Figure 2D**, **Supplementary Figure S1**). PCA indicated that the two clusters can be distinguished clearly (**Figure 2E**).

### Relationships between the clusters with prognosis and clinical features

We further explored whether the two clusters were different in prognosis and clinical features. K-M analysis confirmed that the two clusters were different in OS time (*p* = 0.049, **Figure 2F**). Patients in cluster 1 had worse prognosis than those in cluster 2. No significant difference was observed in the distribution of age between the two clusters, but tumor sizes were found to be different, with the tumor sizes in cluster 1 being larger than those in cluster 2 (*p* < 0.05, **Figure 2G**). Moreover, other clinical features (PAM50 subtype, tumor location, pathologic N, clinical stage, and pathologic grade) were also compared. Doughnut diagrams showed that PAM50 subtype, clinical stage, and pathologic grade were significantly different. As shown in **Figure 2H**, cluster 1 was preferentially associated with higher mortality risk (*p* = 0.095), more threatening PAM50 subtype (*p* < 0.0001), higher pathologic grade (*p* = 0.00098), and higher clinical stage (*p* = 0.039), compared to those in cluster 2. Multi-omics landscape of samples in the two clusters is shown in **Figure 2I**. In contrast to cluster 2, cluster 1 was distinctly related to higher mutation rates and higher CNV rates. Moreover, we noticed that the mutation frequency of TP53 was significantly higher in cluster 1 (*p* = 0.0002, **Supplementary Figure S2**), and the MUC16 was negatively correlated with most of the CAF-related genes (**Supplementary Figure S3**). The results mentioned above verified that two clusters were different in prognosis and clinical features.

### Correlations of the clusters with TME characteristics

**Figure 3:**
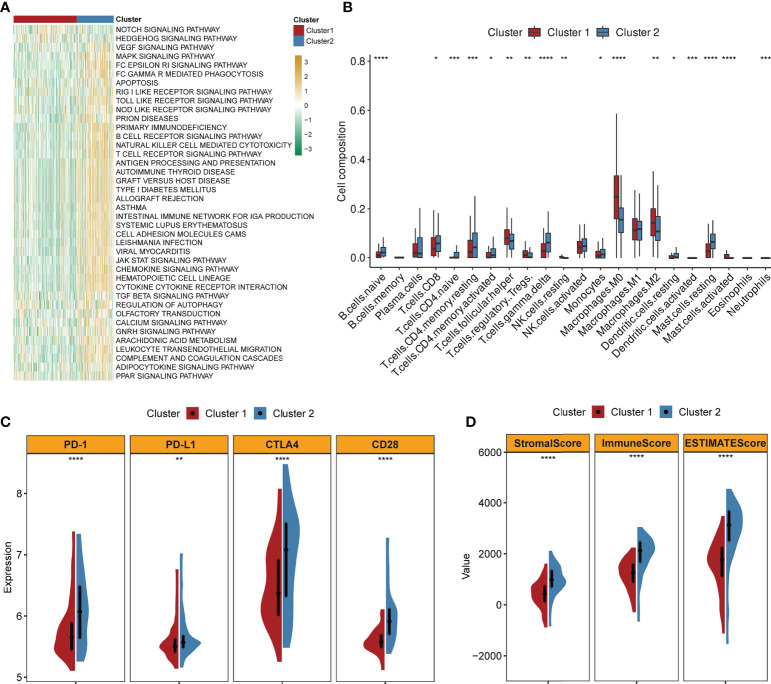
Correlations of clusters with TME characteristics (firebrick: cluster 1; steel blue: cluster 2; ****, ***, **, and * mean p < 0.0001, < 0.001, < 0.01, and < 0.05, respectively). **(A)** Heatmap of GSVA indicates difference in biological processes between two clusters (yellow: upregulated; green: downregulated). **(B)** Abundance of 22 immune cells in two clusters. **(C)** Violin plots of the expression levels of classical immune checkpoints in two clusters. **(D)** Violin plots of the TME scores in two clusters.

GSVA implied that cluster 2 was obviously enriched in immune-activated pathways such as chemokine signaling pathway activation, cytokine receptor interaction, antigen processing and presentation, T- and B-cell receptor signaling pathway, and natural killer cell-mediated cytotoxicity (**Figure 3A**). By applying the CIBERSORT algorithm, the correlations between the two clusters and immune cells of each TNBC patient were assessed. **Figure 3B** confirmed that significant differences were observed in the composition of most immune cells between the two clusters. The composition levels of naive B cells and T cells (CD8+, CD4+, and gamma delta) were visibly higher in cluster 2 than those in cluster 1, while the number of T cells (follicular helper and Tregs), macrophage 0 (M0) cells, and M2 cells was lower in cluster 2 compared to those in cluster 1. Similarly, the expression levels of several important immune checkpoints (PD-1, PD-L1, CTLA4, and CCD28) were significantly higher in cluster 2 (**Figure 3C**). The TME scores (stromal score, immune score, and ESTIMATE score) of the two clusters were also evaluated. **Figure 3D** demonstrates that higher TME scores were observed in cluster 2.

### Identification of gene clusters based on DEGs

**Figure 4:**
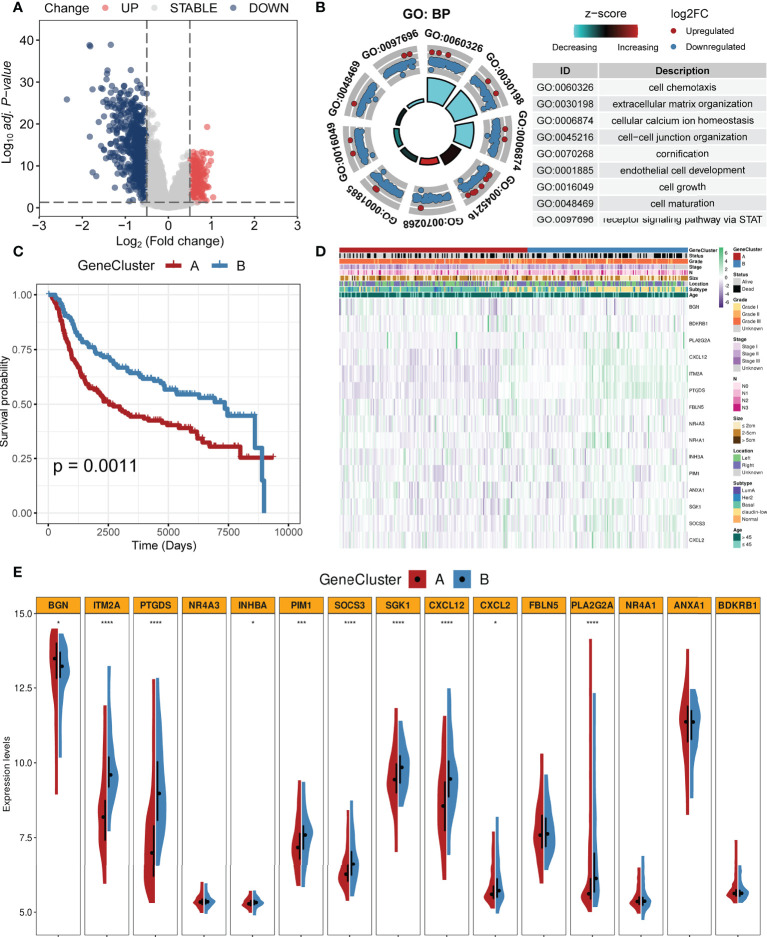
Identification of gene clusters based on the DEGs. **(A)** Volcano plot of DEGs between two clusters (firebrick: upregulated; gray: not change; steel blue: downregulated). **(B)** GO enrichment analysis based on the DEGs. **(C)** K-M survival analysis of two gene clusters (firebrick: gene cluster A; steel blue: gene cluster B). **(D)** Heatmap of two gene clusters with clinical features. **(E)** Violin plots of the expression levels of CAF-related genes in two gene clusters (****, ***, and * mean p < 0.0001, < 0.001, and < 0.05, respectively).

We identified 877 DEGs between the two clusters. Among them, 252 genes were upregulated while 625 genes were downregulated in cluster 1. All DEGs are shown in **Supplementary Table S1**. **Figure 4A** shows these DEGs with a volcano plot. Based on the DEGs, we performed enrichment analysis and the results are shown in **Figure 4B**. Biological processes including cell chemotaxis, extracellular matrix organization, cellular calcium ion homeostasis, cell–cell junction organization, cornification, endothelial cell development, cell growth, cell maturation, and STAT signal pathway were involved. Cox regression analysis was employed to select prognostic DEGs (*p* < 0.05). A CC algorithm was applied for further validation and two genomic clusters based on prognostic DEGs were found, namely, gene cluster A (*n* = 161) and gene cluster B (*n* = 137) (**Supplementary Figure S4**). K-M survival analysis showed that patients in gene cluster A owned worse OS time than those in gene cluster B (*p* = 0.0011, **Figure 4C**). A heatmap with clinical features is shown in **Figure 4D**. In addition, we found that the two gene clusters showed significant differences in the expression of most CAF-related genes (**Figure 4E**).

### Construction of the CPI

**Figure 5:**
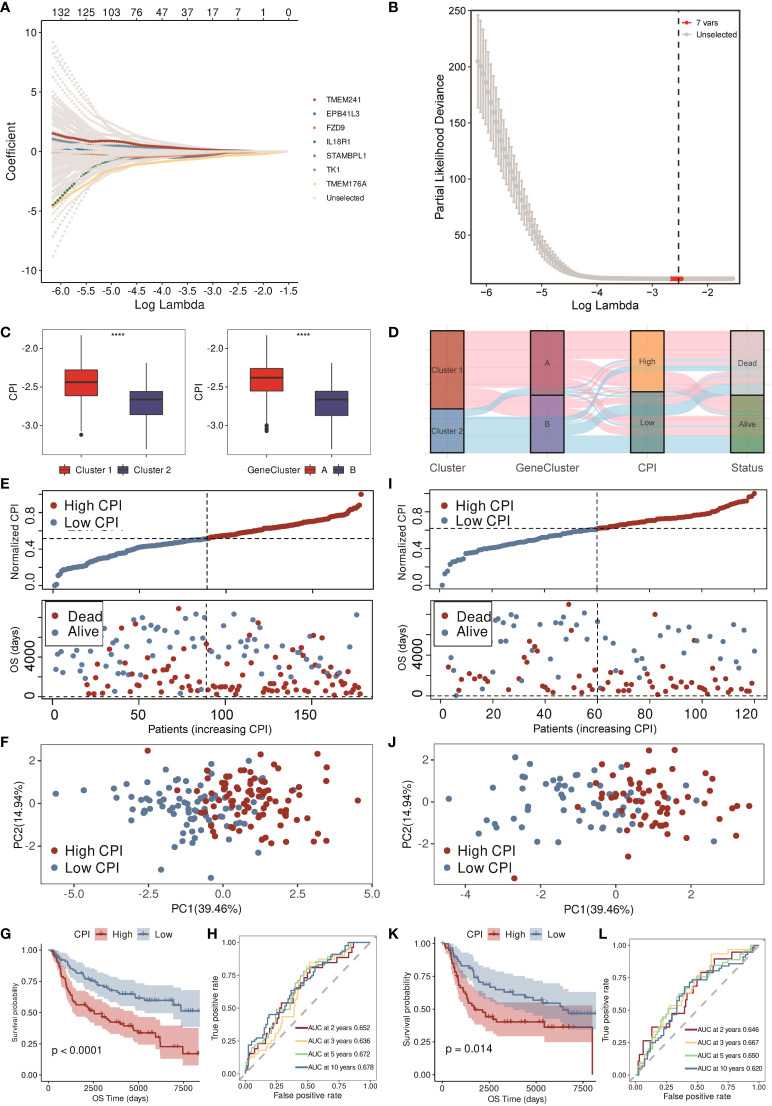
Construction and validation of the CPI in the METABRIC cohort. **(A)** LASSO-Cox regression of the model genes. **(B)** Cross-validation for the LASSO-Cox regression. **(C)** Boxplots of the values of CPI between clusters and gene clusters (**** means p < 0.0001). **(D)** Alluvial diagram of the cluster, gene cluster, CPI group, and survival status. **(E)** Distribution of adjusted CPI and PCA plot based on the CPI groups in the METABRIC training cohort. **(F)** PCA plot in the METABRIC training cohort. **(G)** K-M survival analysis of the patients in the METABRIC training cohort (firebrick: high CPI; steel blue: low CPI). **(H)** ROC curve analysis according to the 2-, 3-, 5-, and 10-year survival of the AUC value in the METABRIC training cohort. **(I)** Distribution of adjusted CPI and PCA plot based on the CPI groups in the METABRIC validation cohort. **(J)** PCA plot in the METABRIC validation cohort. **(K)** K-M survival analysis of the patients in the METABRIC validation cohort (firebrick: high CPI; steel blue: low CPI). **(L)** ROC curve analysis according to the 2-, 3-, 5-, and 10-year survival of the AUC value in the METABRIC validation cohort.

By the LASSO-Cox regression method, a seven-gene (IL18R1, STAMBPL1, EPB41L3, TK1, TMEM176A, TMEM241, and FZD9) signature was constructed in the training cohort (*n* = 178, **Figures 5A, B**). Correlations of each model gene were investigated (**Supplementary Figure S5**). Their respective influence on the OS time was explored by K-M survival analysis (**Supplementary Figure S6A**). Wilcoxon test was applied to explore their expression levels between normal and TNBC tissues in the TCGA cohort (**Supplementary Figure S6B**). All the model genes were significantly related to the prognosis, and the expression of each gene was obviously different except for IL18R1 and TMEM176A (*p* < 0.05). CPI = (−0.070866632 * IL18R1 exp.) + (−0.033907919 * STAMBPL1 exp.) + (−0.007310944 * EPB41L3 exp.) + (0.058986821 * TK1 exp.) + (−0.310459504 * TMEM176A exp.) + (0.054104874 * TMEM241 exp.) + (−0.057525638 * FZD9 exp.). **Figure 5C** shows that higher CPI corresponded with poorer prognosis. The alluvial diagram also indicated that most of the patients in cluster 1 and gene cluster A belonged to the high-CPI group and eventually passed away (**Figure 5D**).

### Training and validation of the CPI

After constructing the CPI, we equally divided 178 TNBC patients in the METABRIC training cohort into low- and high-CPI groups according to the median CPI. **Figure 5E** showed that the survival time of patients became shorter with the increasing CPI, and the PCA showed that the classification was optimal (**Figure 5F**). There was a difference in OS time between these two groups; that is, patients in the high-CPI group were more likely to die (*p* < 0.0001, **Figure 5G**). Moreover, ROC curve analysis implied that the CPI had a favorable predictive efficacy. The area under the ROC curve (AUC) was 0.652 for 2-year, 0.636 for 3-year, 0.672 for 5-year, and 0.678 for 10-year survival (**Figure 5H**). Subsequently, we utilized the METABRIC validation cohort to assess the efficacy of the CPI. The METABRIC validation cohort that contained 120 TNBC patients were also distributed to two groups based on the median CPI. Results showed that high CPI resulted in poor survival time, and these two groups were distinguished satisfactorily (**Figures 5I, J**). K-M survival analysis showed that patients with low CPI were more likely to survive (*p* = 0.014, **Figure 5K**). The AUC was 0.646 for 2-year, 0.667 for 3-year, 0.650 for 5-year, and 0.620 for 10-year survival in the validation cohort (**Figure 5L**).

**Figure 6:**
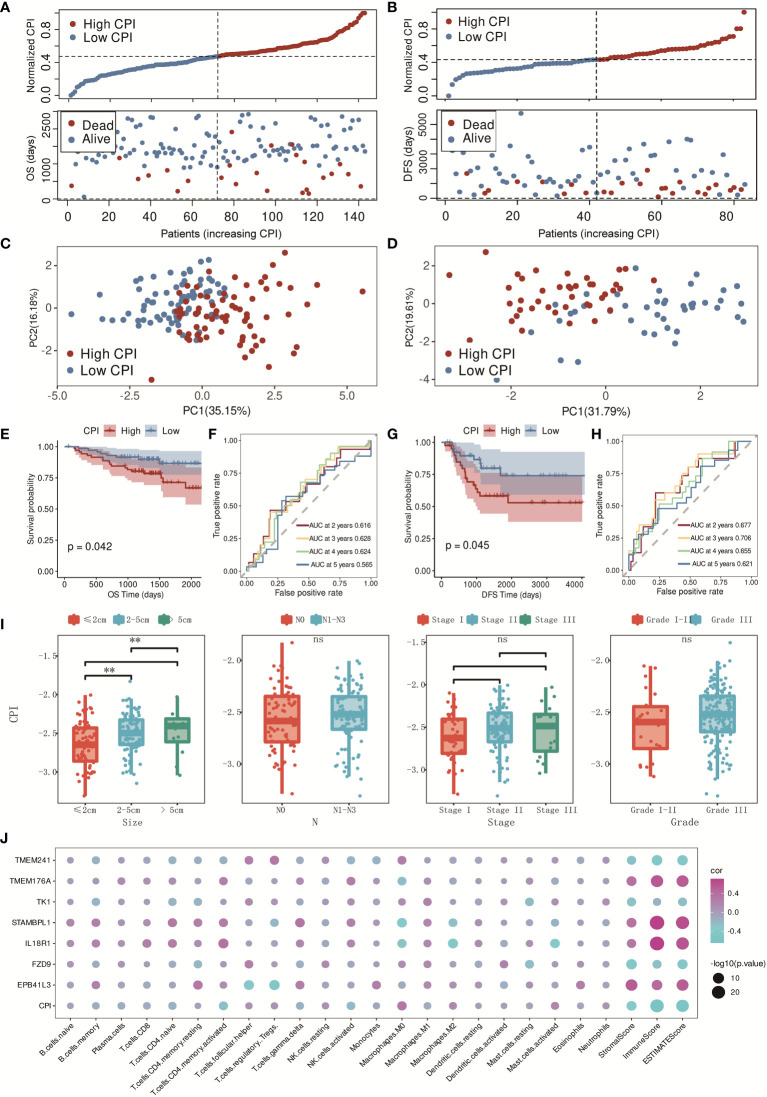
Validation of the CPI in GSE96058 and GSE21653 cohorts. **(A)** Distribution of adjusted CPI based on the CPI groups in the GSE96058 validation cohort. **(B)** Distribution of adjusted CPI based on the CPI groups in the GSE21653 validation cohort. **(C)** PCA plot in the GSE96058 validation cohort (firebrick: high CPI; steel blue: low CPI). **(D)** PCA plot in the GSE21653 validation cohort (firebrick: high CPI; steel blue: low CPI). **(E)** K-M survival analysis of the patients in the GSE96058 validation cohort (firebrick: high CPI; steel blue: low CPI). **(F)** ROC curve analysis according to the 2-, 3-, 4-, and 5-year survival of the AUC value in the GSE96058 validation cohort. **(G)** K-M survival analysis of the patients in the GSE21653 validation cohort (firebrick: high CPI; steel blue: low CPI). **(H)** ROC curve analysis according to the 2-, 3-, 4-, and 5-year survival of the AUC value in the GSE21653 validation cohort. **(I)** Relationships between CPI groups and clinical features (ns means no significance, and ** means p < 0.01). **(J)** Relationships between model genes, CPI, and TME characteristics (purple: positive correlation; blue: negative correlation).

The CPI was also certified in the independent validation cohorts, GSE96058 and GSE21653. Similar distributions with clearly separated PCA plots were found in GSE96058 and GSE21653 (**Figures 6A–D**), and K-M survival analysis indicated that the CPI was a risk factor in TNBC patients (*p* = 0.042 in GSE96058 and *p* = 0.045 in GSE21653, **Figures 6E, G**). The AUC was 0.616 for 2-year, 0.628 for 3-year, 0.624 for 4-year, and 0.565 for 5-year survival in the GSE96058 validation cohort (**Figure 6F**), and the AUC was 0.677 for 2-year, 0.706 for 3-year, 0.655 for 4-year, and 0.621 for 5-year survival in the GSE21653 validation cohort (**Figure 6H**). The CPI was found to be strongly associated with tumor sizes. Higher CPI corresponded with larger tumor sizes. There was no significant difference found between these two groups with other clinical features such as N (N0–N3), stage (I–III), and grade (I–III) (**Figure 6I**). The relationships in the model genes, CPI, and the characteristics of the TME were also identified (**Figure 6J**). In conclusion, CPI showed robust prognostic accuracy in both training and validation cohorts.

### Establishment and assessment of the nomogram model

**Figure 7:**
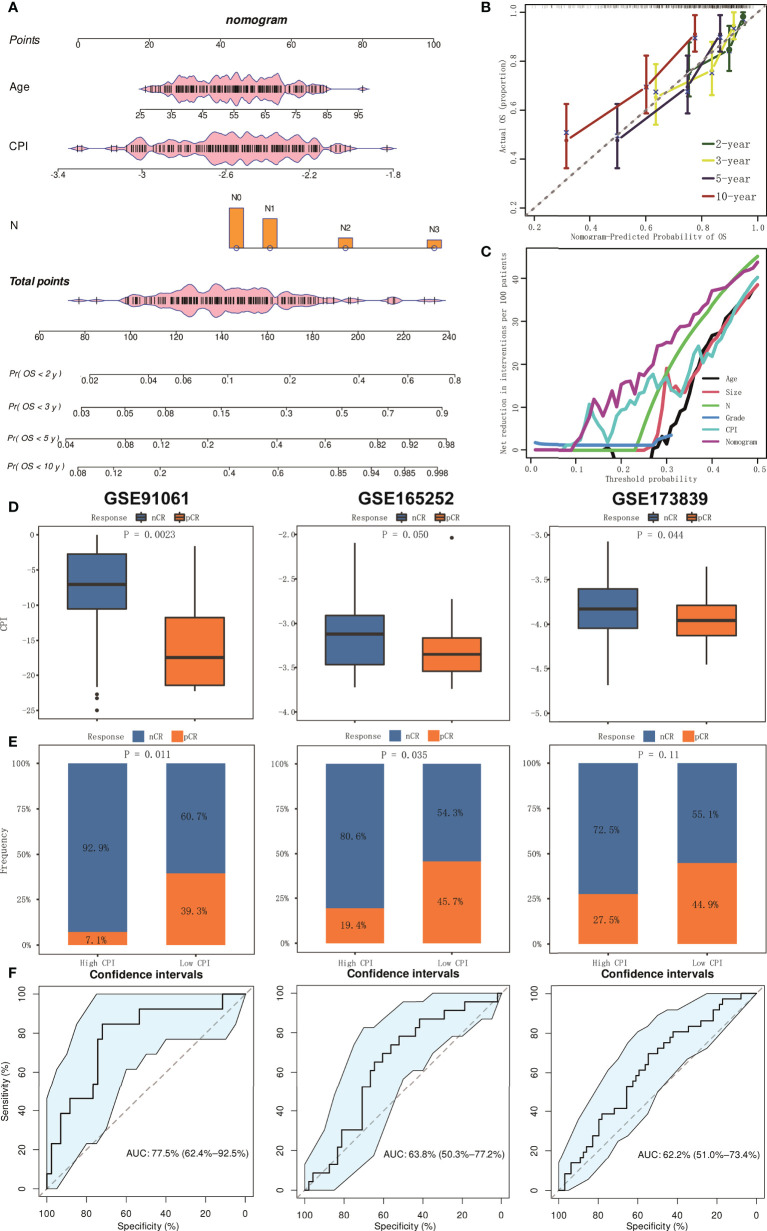
The establishment and assessment of the nomogram and prediction of immunotherapy responsiveness. **(A)** Nomogram based on clinical features and CPI. **(B)** Calibration curves in predicting the 2-, 3-, 5-, and 10-year survival rate. **(C)** DCA of nomogram comparing age, tumor size, pathologic N, grade, and CPI. **(D)** Boxplots of the values of CPI between pCR and nCR groups in GSE91061, GSE165252, and GSE173938 cohorts. **(E)** Results of chi-square test between responsiveness and CPI groups in GSE91061, GSE165252, and GSE173938 cohorts. **(F)** ROC curve analysis of predicting the immunotherapy responsiveness in GSE91061, GSE165252, and GSE173938 cohorts.

Multivariable Cox analyses with stepwise regression were used to establish a nomogram model in the training cohort. Age, CPI, and N were brought into the nomogram model (**Figure 7A**). Calibration curves showed that the accuracy of the nomogram in predicting the 2-, 3-, 5-, and 10-year survival rate was favorable (**Figure 7B**). Moreover, we performed decision curve analysis (DCA), and the result showed that the nomogram was significantly superior to any other variates applied in this study (**Figure 7C**).

### CPI predicts the responsiveness of immunotherapy

We further conducted analysis to explore whether CPI could predict the responsiveness of immunotherapy. We collected three relevant independent cohorts (GSE91061, GSE165252, and GSE173839). Box plots showed that CPI was lower in the pathologic complete response (pCR) group compared to the non-complete response (nCR) group (all *p* < 0.05, **Figure 7D**). Chi-square test also confirmed that the pCR ratio in the low-CPI group was much higher than that of nCR (*p* = 0.011 in GSE91061, *p* = 0.035 in GSE165252, and *p* = 0.11 in GSE173839) (**Figure 7E**). In addition, ROC curve analysis indicated that CPI had excellent efficacy in predicting immunotherapy responsiveness (AUC = 0.775 in GSE91061, 0.638 in GSE165252, and 0.622 in GSE173839) (**Figure 7F**).

## Discussion

Increasing research has suggested that CAFs are vital to the interactions of TME and tumor cells of TNBC (8, 9). Nevertheless, the majority of studies have centralized on a single gene; thus, the overall effect regulated by multiple CAF-related genes has not yet been fully illustrated. In this study, we firstly summarized the CAF-related genes for comprehensive analysis and found a new pattern of CAF-related clusters. A different prognosis was found in CAF-related clusters. Furthermore, these CAF-related clusters were significantly related to clinical and multi-omics features. Cluster 1 was preferentially associated with higher mortality risk, more threatening PAM50 subtype, higher clinical stage, higher pathologic grade, higher mutation rates, and higher CNV rates compared to those in cluster 2. TME characteristics and immune activation also differed obviously between the two clusters, which was consistent with former studies (30, 31). We also identified two gene clusters based on the DEGs between the two CAF-related clusters. Therefore, our study verifies that CAFs could be effective in predicting clinical outcomes and immunotherapy responsiveness in TNBC patients. Then, a seven-gene signature was constructed in the METABRIC cohort, which was then validated to perform well in independent validation cohorts. Patients with high or low CPI showed a significant difference in prognosis, clinical features, and TME characteristics. A nomogram that included clinical features and CPI was built and verified to be effective. Finally, we confirmed that CPI was a predictor for immunotherapy responsiveness.

Among breast cancer patients, patients with TNBC have the worst prognosis. In spite of improvements in immunotherapy, the prognosis of TNBC patients continues to exhibit heterogeneity, which emphasizes the key role of TME in TNBC development and progression (32, 33). Immune cells are the main components of TME, which include lymphocytes, granulocytes, macrophages, and other cells. They are involved in modulating multifarious immune responses, such as the pro-inflammatory response coordinated by tumor cells that can promote survival (34). Stromal cells around tumor cells are also relevant to tumor progression (5, 6). In our study, the CAF-related pattern characterized by cluster 1 (immune inhibition) corresponded with a higher CPI, while cluster 2 (immune activation) was associated with a lower CPI. A growing body of evidence has proven that the differentiation of T cells has a close relationship with TNBC prognosis. Higher infiltration levels of CD4+, CD8+, and gamma delta T cells indicate better prognosis in TNBC patients (35, 36). Cluster 2 and low CPI, which is closely associated with a better prognosis, showed higher levels of activated CD4+, CD8+, and gamma delta T cells, which suggests holding off the development of TNBC. Tregs is a suppressor during the anti-tumor immune response, which is consistent with our finding that the infiltration level of Tregs was higher in cluster 1 with higher CPI (37). It is well known that M1 and M2 cells are opposed to inflammatory-related responses (38). Previous studies also confirmed that the enrichment of M1 cells contributed to a protective factor while the higher proportion of M2 cells was a risk factor in TNBC patients, which corresponds to our results (4, 39).

With the intensive study of molecular biology and tumor immunology, immunotherapy using immune checkpoint inhibitors (ICIs) has been widely accepted (40, 41). Classical immune checkpoints included PD-1, PD-L1, CTLA4, and CCD28. Research targeting these molecules is flourishing and clinical studies have demonstrated their efficacy and safety in the treatment of TNBC (42, 43). In our study, higher expression levels of immune checkpoints were observed in cluster 2 compared to cluster 1. Moreover, independent cohorts confirmed that CPI was significantly associated with immunotherapy responsiveness. Patients with low CPI might benefit from immunotherapy.

There were some limitations in our study. First, sampling bias might exist because of tumor heterogeneity, which causes the different proportion of the interstitial portion in each sample. Second, although the METABRIC cohort is the largest public cohort of breast cancer in the world, it is a type of microarray that might have gaps in the abundance and depth of sequencing. Third, the detailed mechanisms of interaction between CAFs and TNBC cells have not been explored. Finally, multicenter clinical queues are necessary for further analysis and verification.

In conclusion, this new pattern proposed by our study is a practical weapon for TNBC patients, which helps us improve our recognition of the TME of TNBC, predict prognosis of TNBC patients, and offer more reliable immunotherapy strategies in the future.

## Data availability statement

The original contributions presented in the study are included in the article/**Supplementary Material**. Further inquiries can be directed to the corresponding authors.

## Ethics statement

Ethical review and approval was not required for the study on human participants in accordance with the local legislation and institutional requirements. Written informed consent from the patients/participants or patients/participants’ legal guardian/next of kin was not required to participate in this study in accordance with the national legislation and the institutional requirements.

## Author contributions

Research design: XX and MC. Data collection: JX, SZ and YZ. Data analysis: JX, SZ and YZ. Manuscript preparation: JX, SZ, YZ, YT, WT, C-WW, SW, XO and WZ. Manuscript editing: XX and MC. All authors confirm that they contributed to manuscript reviews, critical revision for important intellectual content, and read and approved the final draft for submission. All authors contributed to the article and approved the submitted version.

## Funding

This research was funded by the National Natural Science Foundation of China (81872152, XX).

## Acknowledgments

We thank all the authors who contributed their valuable methods and data and made them public. We thank Dr. Jianming Zeng (University of Macau) and all the members of his bioinformatics team, Biotrainee, for generously sharing their experience and codes. We thank Biotrainee for the use of the biorstudio high-performance computing cluster (https://biorstudio.cloud) and the Shanghai HS Biotech Co., Ltd for conducting the research reported in this paper.

## Conflict of interest

The authors declare that the research was conducted in the absence of any commercial or financial relationships that could be construed as a potential conflict of interest.

## Publisher’s note

All claims expressed in this article are solely those of the authors and do not necessarily represent those of their affiliated organizations, or those of the publisher, the editors and the reviewers. Any product that may be evaluated in this article, or claim that may be made by its manufacturer, is not guaranteed or endorsed by the publisher.
