# Supplementary material for: Turning up a new pattern: Identification of cancer-associated fibroblast-related clusters in TNBC
Source: Front Immunol. 2022 Oct 6;13:1022147. doi: 10.3389/fimmu.2022.1022147 (PMC9583405; doi:10.3389/fimmu.2022.1022147)

Supplementary Figure Legends

Supplementary Figure S1. Unsupervised clustering of the CAF-related genes.

(A) 298 TNBC patients were divided into three or four clusters (k = 3 and 4). **(B)** Cumulative Distribution Function (CDF) Plot under k = 2-8.

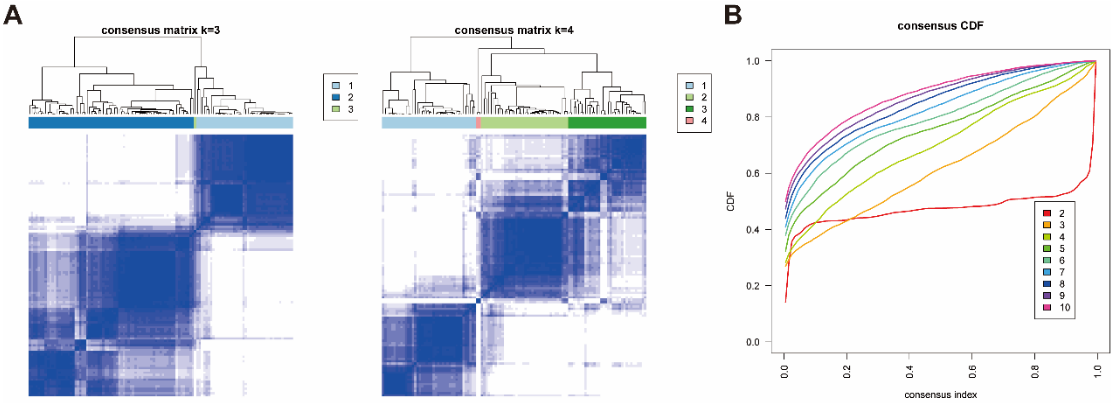

Supplementary Figure S2. Results of chi-square test between TP53 mutation types and the two clusters.

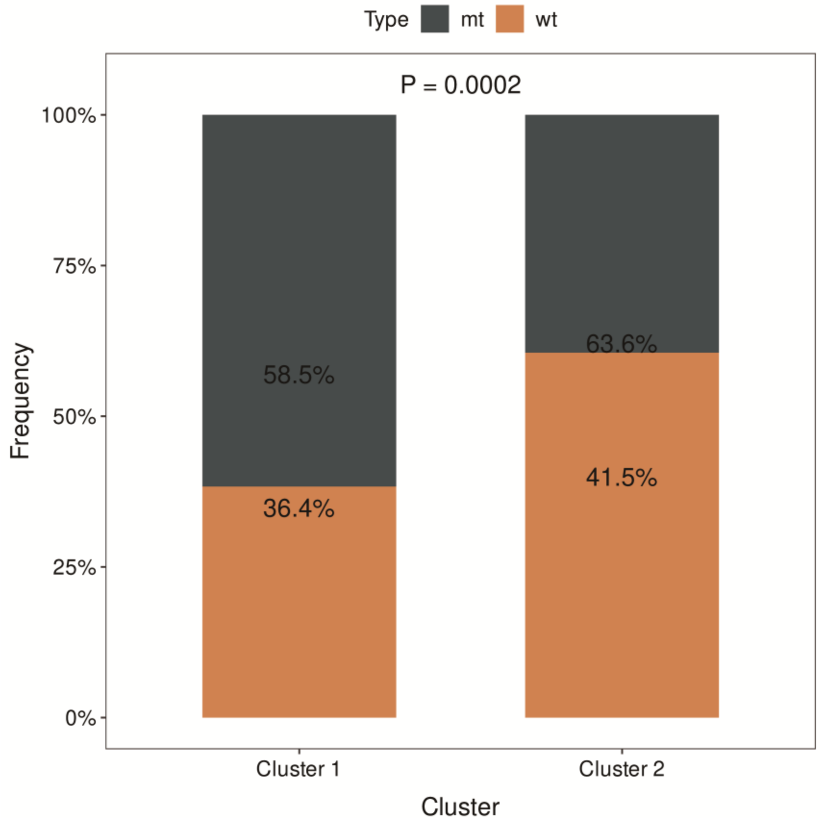

Supplementary Figure S3. Correlation between MUC16 and each CAF-related gene.

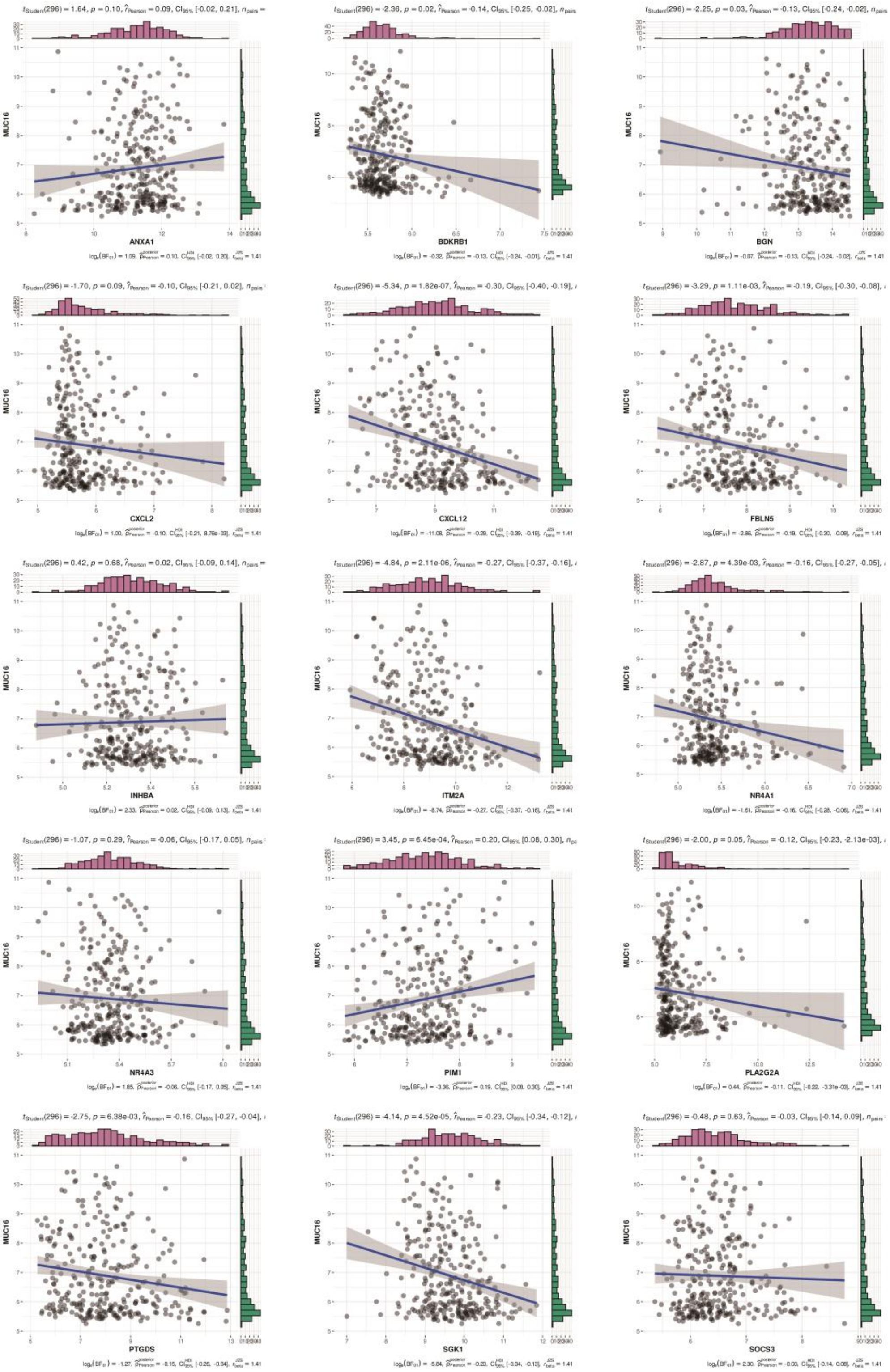

Supplementary Figure S4. Unsupervised clustering of the prognostic DEGs.

(**A**) 298 TNBC patients were divided into two, three, and four gene clusters (k = 2, 3, and 4). (**B**) CDF Plot under k = 2-8.

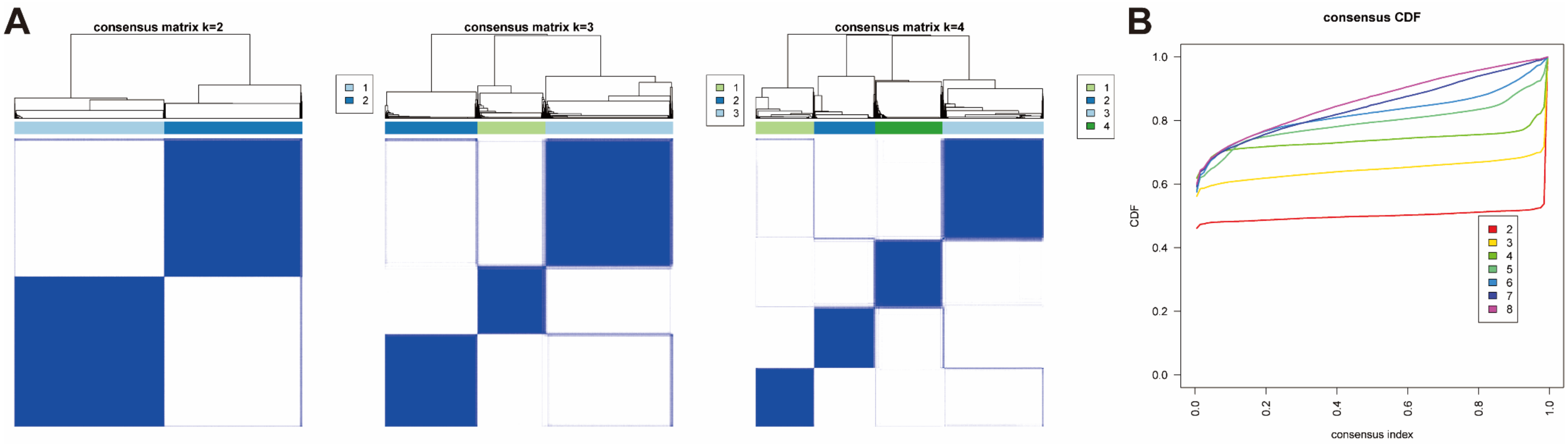

Supplementary Figure S5. Correlation of each model genes.

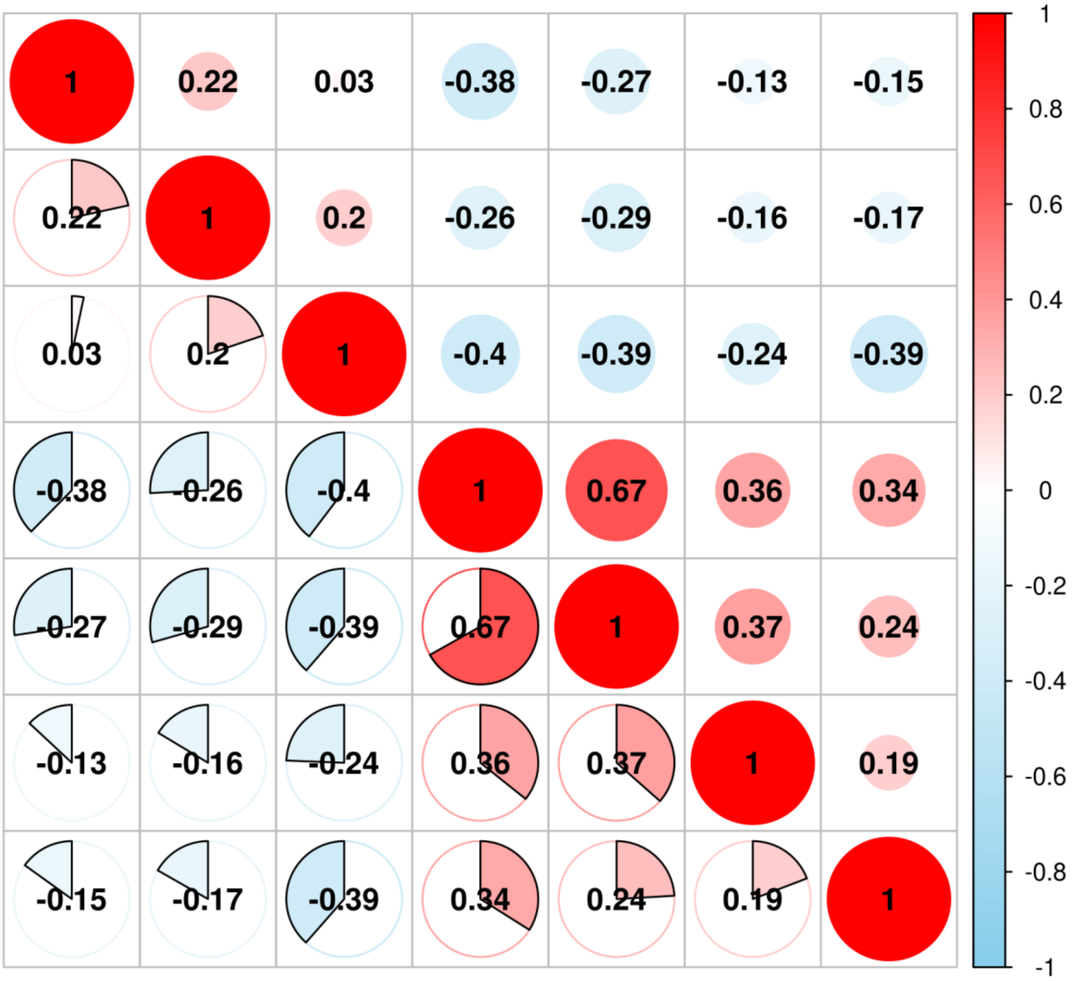

Supplementary Figure S6. The prognostic value and expression levels of each model gene.

(**A**) K-M analysis of each model gene. (**B**) The expression levels of each model gene between TNBC and normal tissues.

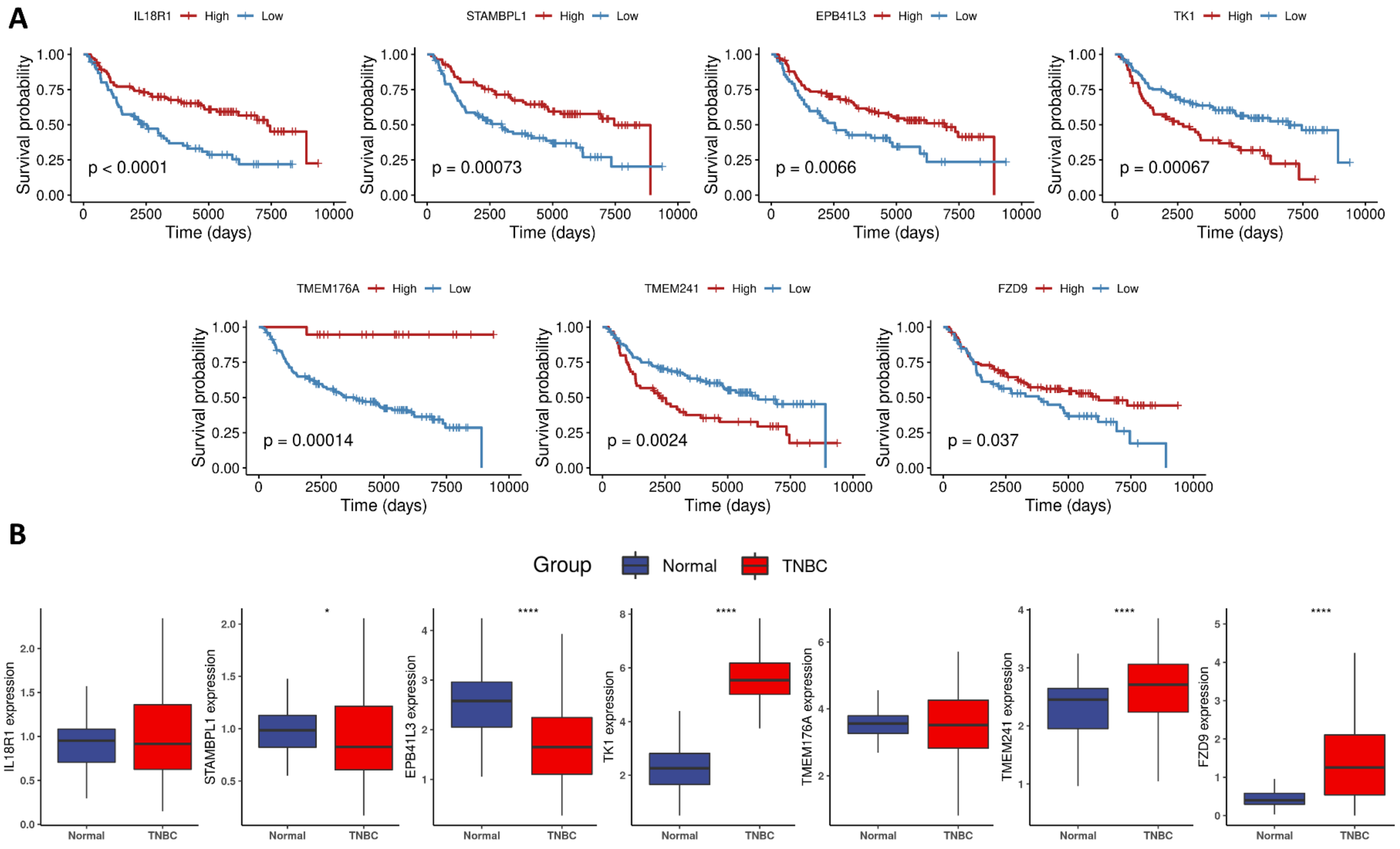

Supplement: Supplementary file 1 [file Image_1.pdf]
