# Supplementary material for: Turning up a new pattern: Identification of cancer-associated fibroblast-related clusters in TNBC
Source: Front Immunol. 2022 Oct 6;13:1022147. doi: 10.3389/fimmu.2022.1022147 (PMC9583405; doi:10.3389/fimmu.2022.1022147)
Supplement: Supplementary file 2 [file Table_1.pdf]

Supplementary Table S1. DEGs between cluster 1 and cluster 2						
Gene	logFC	AveExpr	t	P.Value	adj.P.Val	B
PTGDS	-2.448169567	8.036519934	-19.30651059	1.99E-54	4.85E-50	112.7391855
ABI3BP	-1.335782509	6.852266128	-16.45082038	1.04E-43	1.27E-39	88.47459771
ITM2A	-1.829870065	8.946902351	-16.38244811	1.88E-43	1.53E-39	87.89259962
DARC	-1.812490531	7.136693863	-16.27265213	4.85E-43	2.96E-39	86.95818877
KLRG1	-0.871304504	6.603214164	-14.73168311	2.81E-37	1.37E-33	73.89543759
KLRB1	-1.413062123	7.20206719	-14.689593	4.03E-37	1.64E-33	73.54071975
GIMAP7	-1.423860402	8.373559233	-14.46105853	2.85E-36	9.90E-33	71.61743533
PRKCB	-1.435153082	7.534380326	-14.08181336	7.19E-35	2.19E-31	68.43703201
SLC9A9	-0.728391119	6.655959845	-13.96209212	1.99E-34	5.38E-31	67.43627759
GNG2	-0.652629743	6.155603802	-13.70007483	1.83E-33	4.45E-30	65.25210841
GIMAP6	-1.023157088	7.707380671	-13.62474839	3.45E-33	7.64E-30	64.62582031
CD37	-1.309933091	7.301009881	-13.60296756	4.15E-33	8.42E-30	64.44486967
AMICA1	-1.032997539	7.178267113	-13.57109571	5.42E-33	1.02E-29	64.18020171
CXCL12	-1.561049727	9.051753025	-13.44505578	1.57E-32	2.73E-29	63.13493989
S1PR1	-0.713296981	6.43294552	-13.41913162	1.95E-32	3.02E-29	62.9202296
AK090694	-0.627479936	6.629631532	-13.41702787	1.98E-32	3.02E-29	62.90281011
CCL15	-1.527184812	7.008673315	-13.36058451	3.19E-32	4.57E-29	62.4356903
GZMK	-1.760473008	7.901226796	-13.33023933	4.12E-32	5.57E-29	62.18475216
PVRIG	-1.228465603	6.719796121	-13.29153964	5.69E-32	7.30E-29	61.86492818
FYN	-0.879642662	7.818648364	-13.23598983	9.07E-32	1.11E-28	61.40625106
IL33	-1.030492864	6.464778594	-13.18531386	1.39E-31	1.61E-28	60.98823622
IRF8	-1.336142973	8.787216065	-13.09101964	3.05E-31	3.38E-28	60.21151198
ABCA6	-0.585461393	5.967248508	-13.07083777	3.61E-31	3.83E-28	60.04545635
TGFBR2	-1.106502644	9.085153165	-13.06314942	3.85E-31	3.91E-28	59.98221455
ARHGAP15	-1.112447013	7.966990305	-13.03750568	4.77E-31	4.65E-28	59.77134828
ABCB1	-0.501539172	6.017125	-12.99979333	6.54E-31	6.13E-28	59.46144205
LRMP	-0.91387311	6.524775584	-12.96037867	9.08E-31	8.20E-28	59.13780305
IL10RA	-0.951619737	7.330483392	-12.94683139	1.02E-30	8.85E-28	59.02662559
CD69	-1.149625007	6.974094705	-12.78663337	3.86E-30	3.24E-27	57.71436499
SAMD3	-0.755385762	6.250507298	-12.70056006	7.88E-30	6.40E-27	57.01119432
CELF2	-0.76516669	6.311104729	-12.67046363	1.01E-29	7.94E-27	56.76564561
ARHGAP9	-1.129698007	8.056639941	-12.65126113	1.18E-29	9.02E-27	56.609066
CCL19	-2.355458426	8.596139013	-12.59296419	1.92E-29	1.42E-26	56.13413239
ENPP2	-1.280097036	7.577133136	-12.5662186	2.39E-29	1.72E-26	55.91645859
KLF2	-1.012814783	9.900647988	-12.56219927	2.48E-29	1.72E-26	55.88375846
TCRA	-1.438036791	7.680535729	-12.55353857	2.66E-29	1.80E-26	55.81330811
GIMAP5	-1.131226409	7.739464087	-12.505259	3.96E-29	2.61E-26	55.42084606
EBF1	-0.828400627	6.996745555	-12.49902204	4.17E-29	2.67E-26	55.37017945
CST7	-0.950899358	6.992290951	-12.45515238	5.99E-29	3.74E-26	55.01401706
MFAP4	-1.511137331	8.119367772	-12.39495067	9.83E-29	5.93E-26	54.52588706
PYHIN1	-0.827222903	6.554902053	-12.39315209	9.98E-29	5.93E-26	54.51131496
PPP1R16B	-1.0819245	6.825763956	-12.38687894	1.05E-28	6.10E-26	54.46049522
WIPF1	-0.865998082	7.525578479	-12.31043461	1.97E-28	1.12E-25	53.84185645
IL7R	-1.41295075	7.963189451	-12.29133582	2.30E-28	1.28E-25	53.68748527
FAIM3	-1.358759078	7.496529191	-12.28653718	2.40E-28	1.30E-25	53.6487109
DOCK10	-0.845350392	7.951181498	-12.26587134	2.84E-28	1.50E-25	53.4817806
STAT4	-0.979407427	7.068076749	-12.24955026	3.25E-28	1.68E-25	53.35000907
BU935198	-0.861821004	7.341607888	-12.23617042	3.62E-28	1.84E-25	53.24202642
RAB37	-0.779511658	6.118786037	-12.23124258	3.77E-28	1.88E-25	53.20226559
ITK	-1.04641296	6.949010499	-12.20442285	4.70E-28	2.29E-25	52.98595871
SELL	-1.272974977	6.749648977	-12.19368805	5.13E-28	2.45E-25	52.89942347
TCRBV4S1A1T	-0.652061822	5.866643715	-12.15995203	6.76E-28	3.13E-25	52.62763303
EVI2B	-1.186268771	8.001937583	-12.15906927	6.81E-28	3.13E-25	52.62052444
GMFG	-1.036224581	8.295907064	-12.14822395	7.44E-28	3.36E-25	52.53320485
DOCK2	-1.055624318	8.591142556	-12.07371967	1.37E-27	6.06E-25	51.93404197
SH2D1A	-0.770139714	6.533080714	-12.05062916	1.65E-27	7.19E-25	51.74859796
RAC2	-1.314262812	9.491320005	-12.04844778	1.68E-27	7.19E-25	51.73108509
INPP5D	-0.712776463	6.829698781	-12.03172942	1.93E-27	8.03E-25	51.59689982
CTSG	-1.410379561	6.572166288	-12.03058716	1.94E-27	8.03E-25	51.58773401
TRAT1	-0.67625237	6.059337728	-12.02196061	2.09E-27	8.47E-25	51.51852225
PECAM1	-0.842672695	8.111946833	-12.01368519	2.23E-27	8.91E-25	51.45214343

LY9	-0.693342873	5.93614567	-11.99891328	2.52E-27	9.89E-25	51.33369347
ADAM7	-0.584845963	6.01794296	-11.93881387	4.10E-27	1.57E-24	50.85229448
HCST	-1.237200296	10.09212796	-11.93797798	4.13E-27	1.57E-24	50.84560483
BANK1	-1.141482285	6.321034157	-11.9329847	4.30E-27	1.61E-24	50.80564682
ARHGAP25	-0.911352808	7.406456935	-11.91754659	4.88E-27	1.80E-24	50.68214194
GPSM3	-0.874845831	7.303403111	-11.8484832	8.55E-27	3.11E-24	50.13031663
GPR18	-0.771038858	6.038177027	-11.81453153	1.13E-26	3.97E-24	49.85945129
GIMAP4	-1.112055903	9.578390943	-11.80601363	1.21E-26	4.20E-24	49.79153893
PIK3IP1	-0.799842496	8.191834989	-11.80395753	1.23E-26	4.21E-24	49.77514841
SH2D3C	-0.78240189	7.343665825	-11.78383641	1.44E-26	4.82E-24	49.61480373
FOLR2	-1.158106086	6.969159565	-11.74459697	1.98E-26	6.53E-24	49.30238671
PRDM8	-0.540023262	6.209967811	-11.73197742	2.19E-26	7.13E-24	49.2019917
PTPRCAP	-1.060245553	6.959399168	-11.72651564	2.29E-26	7.36E-24	49.15855246
EVI2A	-0.924778423	7.152922824	-11.65891953	3.96E-26	1.25E-23	48.62154727
BTLA	-0.562173383	5.908259609	-11.65425677	4.11E-26	1.28E-23	48.58454651
CH25H	-0.935693336	6.484273272	-11.62568392	5.17E-26	1.58E-23	48.35792886
P2RY10	-0.752572176	6.114568992	-11.57509658	7.78E-26	2.34E-23	47.95721315
LYL1	-0.866442036	8.418607976	-11.53828972	1.05E-25	3.11E-23	47.66606505
WAS	-1.012650867	9.062155426	-11.53180386	1.10E-25	3.23E-23	47.61479679
SLAMF1	-0.7244043	6.139047869	-11.50824361	1.33E-25	3.86E-23	47.4286533
CHST7	-0.579937281	7.246721584	-11.49964655	1.43E-25	4.09E-23	47.36076586
GPR183	-1.10755814	7.885586216	-11.42401199	2.61E-25	7.24E-23	46.76434176
S1PR4	-0.623485784	6.078920719	-11.41524088	2.80E-25	7.67E-23	46.69527362
ZNF831	-0.596661852	5.888716406	-11.40297081	3.09E-25	8.29E-23	46.59868706
HCLS1	-1.098703596	9.450993536	-11.40062127	3.15E-25	8.35E-23	46.58019674
LRRC33	-0.704762637	6.797158287	-11.39866618	3.20E-25	8.39E-23	46.5648117
CCR7	-1.291521196	6.944309559	-11.37820685	3.77E-25	9.69E-23	46.40387414
GRAP	-0.815903447	7.485244872	-11.37793941	3.78E-25	9.69E-23	46.4017711
PIP4K2A	-0.743784009	9.84471708	-11.35855553	4.41E-25	1.12E-22	46.2493979
TMEM119	-1.238487905	8.786872245	-11.35361241	4.59E-25	1.15E-22	46.21055708
ABCA8	-0.756916837	6.113560595	-11.33341026	5.39E-25	1.34E-22	46.05188592
CD5	-0.64576987	6.080351807	-11.33127348	5.48E-25	1.35E-22	46.03510969
TCRVB	-1.49348196	8.394818941	-11.31071922	6.46E-25	1.57E-22	45.87379826
KLHL6	-0.713804667	6.381417133	-11.30498224	6.76E-25	1.63E-22	45.82879448
TEK	-0.662735201	6.753672749	-11.30298276	6.87E-25	1.64E-22	45.81311164
CD48	-1.21567434	7.593859414	-11.28572509	7.88E-25	1.86E-22	45.67779715
JAM2	-0.654195442	6.548684298	-11.27365532	8.68E-25	2.03E-22	45.5832085
BTG2	-0.970914425	8.212111316	-11.26997856	8.94E-25	2.07E-22	45.55440234
CD1C	-0.762520173	6.280379643	-11.24863414	1.06E-24	2.43E-22	45.38724945
LSP1	-0.666376064	6.388710228	-11.13495451	2.61E-24	5.89E-22	44.49913411
RNASE6	-0.903511289	7.393184196	-11.12012704	2.94E-24	6.57E-22	44.38356368
DPEP2	-0.728885506	6.451089598	-11.06565166	4.52E-24	1.00E-21	43.95950284
PLAC8	-1.180312312	6.864282348	-11.05489538	4.92E-24	1.08E-21	43.87587203
ZEB2	-0.649598416	6.589392486	-11.02294162	6.33E-24	1.38E-21	43.62762705
IGF1	-0.579095023	5.721346275	-11.02144469	6.41E-24	1.38E-21	43.61600481
DNASE1L3	-0.818273356	5.949907349	-11.01452578	6.77E-24	1.45E-21	43.5622948
ELMO1	-0.715636216	7.25478759	-11.00818073	7.12E-24	1.51E-21	43.51305173
PAG1	-0.624146534	6.836879039	-10.97539944	9.21E-24	1.94E-21	43.25882894
NAPSB	-1.186991827	7.633206639	-10.95920851	1.05E-23	2.16E-21	43.13338302
TSPAN7	-1.097401111	6.771152198	-10.95920051	1.05E-23	2.16E-21	43.13332109
RGS18	-0.573339792	6.257017402	-10.95254959	1.10E-23	2.26E-21	43.08181277
SCARA5	-0.717554111	5.805480006	-10.91640716	1.47E-23	2.98E-21	42.80213632
P2RY8	-0.996448529	6.821257381	-10.89874531	1.68E-23	3.36E-21	42.66560759
DOCK11	-0.752695798	7.41604842	-10.89487549	1.74E-23	3.44E-21	42.63570575
CD52	-1.616286591	9.136415593	-10.89225618	1.77E-23	3.48E-21	42.61546917
CD247	-1.270925413	8.189635643	-10.84043395	2.66E-23	5.19E-21	42.21551895
CCR6	-0.616353755	6.011644128	-10.83717031	2.73E-23	5.28E-21	42.1903583
MAN1C1	-0.847394175	6.756620642	-10.82148758	3.09E-23	5.92E-21	42.0694993
TBC1D10C	-1.106657486	7.316801303	-10.80810767	3.43E-23	6.53E-21	41.96644642
APBB1IP	-0.812591243	8.168873837	-10.78562353	4.09E-23	7.72E-21	41.79339585
AKNA	-0.874454213	7.763020346	-10.71422212	7.14E-23	1.32E-20	41.24488675
PLCL2	-0.723204871	7.599901476	-10.6873072	8.81E-23	1.61E-20	41.03853786
COL14A1	-0.729038594	6.38037567	-10.63411349	1.33E-22	2.42E-20	40.63138955

CORO1A	-0.986876277	8.080487812	-10.62554779	1.42E-22	2.53E-20	40.56591119
PGM5	-0.864046893	6.465023158	-10.62548307	1.42E-22	2.53E-20	40.56541655
CST3	-0.878242105	11.66990341	-10.61273724	1.57E-22	2.78E-20	40.46802778
LDB2	-0.776497196	7.652997032	-10.60389926	1.68E-22	2.95E-20	40.40052882
CFD	-1.367038054	8.702225526	-10.57872562	2.05E-22	3.54E-20	40.20840582
ALDH1A1	-1.126119679	7.538270803	-10.56235699	2.33E-22	3.99E-20	40.08359157
CLECL1	-0.736951741	6.342480342	-10.55795874	2.41E-22	4.10E-20	40.05006877
FCRH3	-0.777668026	6.118649357	-10.55436964	2.47E-22	4.19E-20	40.02271788
BAIAP2L1	0.901713563	8.459391971	10.5262314	3.08E-22	5.17E-20	39.80843394
CD8A	-1.234376447	7.790991345	-10.50820184	3.54E-22	5.90E-20	39.67126748
RGL1	-0.694737207	8.949719903	-10.49711885	3.85E-22	6.39E-20	39.58700245
CXCR5	-1.009829841	6.077765356	-10.48013497	4.39E-22	7.24E-20	39.4579507
SVEP1	-1.072893966	7.552915252	-10.47508576	4.57E-22	7.47E-20	39.41960268
CXCR3	-0.815617466	6.455952655	-10.43816593	6.08E-22	9.80E-20	39.13945848
C5orf20	-0.662441982	6.291897625	-10.40603994	7.78E-22	1.24E-19	38.89605864
PLAC9	-1.171905685	7.426500298	-10.3946665	8.49E-22	1.34E-19	38.80997149
LPXN	-0.888666579	9.367932581	-10.38630283	9.06E-22	1.42E-19	38.7466935
PTGER4	-0.667275056	7.429160241	-10.37336101	1.00E-21	1.55E-19	38.64882448
MEOX1	-0.726777885	6.190697047	-10.3720332	1.01E-21	1.56E-19	38.63878643
MAP4K1	-1.017205255	7.610500676	-10.35272985	1.17E-21	1.80E-19	38.49292381
CFH	-0.860218713	7.01139033	-10.33593759	1.33E-21	2.03E-19	38.36613861
VAMP5	-0.846538408	9.426751606	-10.32609098	1.44E-21	2.17E-19	38.29183922
LHFP	-0.824421984	7.849936718	-10.32596434	1.44E-21	2.17E-19	38.29088384
ADRB2	-0.634130711	6.484244612	-10.32450422	1.46E-21	2.18E-19	38.2798691
CYSLTR1	-0.574533611	6.369866793	-10.32370811	1.47E-21	2.18E-19	38.27386381
CD2	-1.293280495	8.421453294	-10.32145945	1.49E-21	2.20E-19	38.25690254
GAPT	-0.754971305	6.646863965	-10.31851047	1.52E-21	2.23E-19	38.23466157
RAB8B	-0.687841945	8.034337548	-10.31232133	1.60E-21	2.32E-19	38.18799322
RGL4	-0.529267229	6.044210375	-10.28113214	2.03E-21	2.93E-19	37.95301523
PTPRC	-0.590506177	6.024489472	-10.27821875	2.08E-21	2.98E-19	37.93108296
TNFSF13	-0.600387389	7.64987057	-10.27145424	2.19E-21	3.12E-19	37.88017035
FLRT2	-0.550267097	6.086103067	-10.26909068	2.23E-21	3.16E-19	37.86238491
GZMA	-1.193308529	7.638204257	-10.2595514	2.40E-21	3.36E-19	37.79062285
TNFAIP8	-0.663405037	6.673433769	-10.25684998	2.45E-21	3.41E-19	37.7703063
CD3D	-1.369780215	8.55490092	-10.2364937	2.86E-21	3.96E-19	37.61729461
TRAF3IP3	-0.867046297	6.850783073	-10.20036277	3.77E-21	5.18E-19	37.34606506
CLEC14A	-0.683249096	7.222133094	-10.17942442	4.42E-21	6.05E-19	37.18909303
ARHGAP30	-0.696110443	7.135871849	-10.16479499	4.94E-21	6.72E-19	37.07950964
C1orf162	-0.844970958	9.509800427	-10.12661107	6.60E-21	8.94E-19	36.79384532
SLC40A1	-1.19606998	8.675419335	-10.09283399	8.54E-21	1.14E-18	36.54158213
PARVG	-0.753666745	7.628828748	-10.08444027	9.10E-21	1.20E-18	36.47895723
TXNIP	-0.798576339	9.861386555	-10.07864923	9.51E-21	1.25E-18	36.4357654
C7	-0.70443645	5.750084774	-10.07595463	9.70E-21	1.27E-18	36.41567219
ZAP70	-0.730605303	6.217490265	-10.043808	1.24E-20	1.61E-18	36.1761611
CD27	-0.933894522	6.889813727	-10.02571267	1.42E-20	1.84E-18	36.04150475
SPARCL1	-1.198383686	9.783172862	-10.02064912	1.48E-20	1.90E-18	36.00384568
MAF	-0.778225112	7.50716285	-10.01934911	1.49E-20	1.91E-18	35.99417864
CLDN5	-0.898774894	6.898354702	-10.01158292	1.58E-20	2.02E-18	35.93644103
PSTPIP1	-0.59043708	6.29718849	-10.00597398	1.65E-20	2.09E-18	35.89475514
TLR10	-0.530255922	5.793767268	-9.95848447	2.36E-20	2.98E-18	35.54227232
RASSF5	-0.838953219	8.066038751	-9.956199703	2.40E-20	3.02E-18	35.52533491
ACAP1	-0.883187735	6.971099948	-9.952800168	2.46E-20	3.08E-18	35.50013708
RASGRP2	-0.596373549	5.83804675	-9.916855375	3.23E-20	4.00E-18	35.23397067
KCTD12	-0.712745756	8.277804866	-9.857734974	5.04E-20	6.20E-18	34.79723793
EMP3	-0.756484657	9.744440363	-9.852027784	5.26E-20	6.44E-18	34.75514725
IL6ST	-0.669455926	8.114898753	-9.848347267	5.41E-20	6.59E-18	34.72800985
CLEC3B	-0.684315334	6.157771171	-9.847504699	5.44E-20	6.59E-18	34.72179809
EOMES	-0.92772504	6.978815513	-9.843661213	5.60E-20	6.75E-18	34.69346574
CNRIP1	-0.506804515	6.085076326	-9.839936916	5.76E-20	6.91E-18	34.66601731
KLRD1	-0.569439141	6.367962236	-9.832426802	6.09E-20	7.28E-18	34.61068296
SAMSN1	-0.667578186	6.719218772	-9.830525138	6.18E-20	7.34E-18	34.59667493
FOXN3	-0.521737412	8.746247249	-9.821947783	6.59E-20	7.79E-18	34.53350944
SDPR	-0.540234135	6.224921006	-9.821363369	6.62E-20	7.79E-18	34.5292067

CD53	-0.75948062	7.128761843	-9.809573398	7.23E-20	8.47E-18	34.44243091
GJA4	-0.617782583	6.94930535	-9.805528598	7.45E-20	8.69E-18	34.41267279
PDE7B	-0.534348447	6.1976501	-9.798647325	7.85E-20	9.10E-18	34.36206067
DOCK8	-0.709493849	7.16231501	-9.787083979	8.55E-20	9.88E-18	34.27705224
IGJ	-1.823267579	8.267150585	-9.771399328	9.62E-20	1.11E-17	34.16182742
CD3G	-0.726346864	6.395138418	-9.769673847	9.74E-20	1.11E-17	34.14915719
TCL1A	-0.76395469	5.825428691	-9.754415192	1.09E-19	1.24E-17	34.03716232
ISCU	-0.513798256	10.0122095	-9.738981562	1.23E-19	1.38E-17	33.92397413
FGD3	-0.81783332	8.112947325	-9.730408131	1.31E-19	1.47E-17	33.86113734
BASP1	-0.909711803	10.30910233	-9.725486734	1.36E-19	1.51E-17	33.82508003
RAMP3	-0.601080416	6.761309642	-9.695837756	1.69E-19	1.88E-17	33.60805082
PDE1A	-0.573415816	6.404362094	-9.690916672	1.75E-19	1.94E-17	33.5720617
FOXO1	-0.590990505	7.480975441	-9.666268874	2.11E-19	2.32E-17	33.39194805
SCARNA9	-0.61912314	6.481631855	-9.62847348	2.79E-19	3.06E-17	33.11621996
HLA-DOA	-0.937143098	8.268153657	-9.621084068	2.95E-19	3.22E-17	33.06237771
HLA-DPA1	-1.067290536	11.62385587	-9.620311658	2.96E-19	3.22E-17	33.05675086
PRF1	-0.774939191	6.551327542	-9.617884932	3.02E-19	3.27E-17	33.03907418
DHRS9	-1.114602645	6.782905785	-9.608198577	3.24E-19	3.49E-17	32.96854032
IFFO1	-0.707161857	8.107541404	-9.607829862	3.25E-19	3.49E-17	32.96585615
PI16	-1.052397386	6.294027066	-9.603342616	3.36E-19	3.58E-17	32.93319424
TNFRSF13B	-0.753952608	6.036423204	-9.594365306	3.59E-19	3.80E-17	32.86787386
PTPRE	-0.603585376	7.825585712	-9.593667536	3.61E-19	3.81E-17	32.86279811
SASH3	-0.784171682	7.255187132	-9.56491817	4.47E-19	4.69E-17	32.65383616
SH2B3	-0.637408823	8.394272738	-9.555724477	4.78E-19	5.00E-17	32.58708214
FCGRT	-0.753557149	9.399090743	-9.53912569	5.40E-19	5.61E-17	32.46664638
FGL2	-0.950377195	8.243374775	-9.538995299	5.41E-19	5.61E-17	32.46570074
C7orf58	-0.634823346	6.906550565	-9.501079953	7.15E-19	7.39E-17	32.19101511
RASAL3	-0.982280158	8.236336719	-9.495940094	7.43E-19	7.64E-17	32.1538229
ST3GAL5	-0.685602917	7.439330509	-9.492144016	7.64E-19	7.82E-17	32.12636118
TPK1	-0.547168451	6.836930669	-9.449329554	1.05E-18	1.07E-16	31.8170358
CD79B	-1.214889839	7.960384504	-9.441911949	1.11E-18	1.12E-16	31.76352095
CD6	-0.995425405	7.799758791	-9.425153835	1.25E-18	1.26E-16	31.64270107
CYTIP	-0.604493526	6.596079028	-9.421474422	1.28E-18	1.29E-16	31.61618924
CPVL	-0.962857896	8.870136304	-9.41155487	1.38E-18	1.39E-16	31.54474203
EBF3	-0.541204155	6.16919092	-9.409008645	1.41E-18	1.41E-16	31.52640893
HSD11B1	-0.629735246	6.339779955	-9.379433704	1.75E-18	1.72E-16	31.31366165
LPAR1	-0.787392262	7.608580584	-9.376850389	1.78E-18	1.74E-16	31.29509571
GNG11	-0.862581056	8.086633307	-9.374859367	1.81E-18	1.76E-16	31.28078839
CD19	-1.225091813	6.672415154	-9.346497134	2.22E-18	2.16E-16	31.0771579
IL18R1	-0.518855122	6.319324923	-9.331044154	2.49E-18	2.41E-16	30.96635176
SLA	-0.578077766	6.893478271	-9.323917243	2.62E-18	2.53E-16	30.91528152
VWF	-0.782226816	10.32006272	-9.320345588	2.69E-18	2.58E-16	30.88969562
NCF4	-0.636455399	6.934654568	-9.30195278	3.08E-18	2.93E-16	30.75802141
LOC643733	-0.707342154	6.935862785	-9.283536085	3.52E-18	3.34E-16	30.62631829
IGFBP4	-0.962302858	10.05042859	-9.281365127	3.58E-18	3.38E-16	30.61080254
BLK	-0.598063858	5.743766863	-9.273551514	3.79E-18	3.56E-16	30.55497536
PRKCH	-0.700102635	8.387242202	-9.23895421	4.87E-18	4.56E-16	30.30809215
LY96	-0.780918876	9.35887557	-9.222246632	5.50E-18	5.07E-16	30.18905002
DCN	-1.335841424	8.217484218	-9.217930448	5.67E-18	5.22E-16	30.15831633
CSF2RA	-0.623643347	6.956442545	-9.208266675	6.08E-18	5.57E-16	30.08953356
CCL21	-1.342504668	7.02781707	-9.206141647	6.18E-18	5.62E-16	30.07441381
TRANK1	-0.592720157	7.304945526	-9.199928173	6.46E-18	5.86E-16	30.03021553
CD4	-0.556494458	6.710712827	-9.196872779	6.61E-18	5.96E-16	30.00848765
CD3E	-0.631643724	6.211494119	-9.161159016	8.56E-18	7.70E-16	29.75481237
ARHGAP4	-0.699357156	7.798446427	-9.154703229	8.97E-18	8.03E-16	29.70901532
LAT	-0.523208391	6.4040484	-9.139583001	1.00E-17	8.86E-16	29.60182345
TLR8	-0.677646367	6.476534233	-9.138587721	1.01E-17	8.89E-16	29.59477107
LEF1	-0.655532753	7.168148142	-9.134411577	1.04E-17	9.10E-16	29.56518432
AOX1	-0.596797522	6.05038252	-9.125437937	1.11E-17	9.67E-16	29.50163425
GVIN1	-1.045924767	7.579996003	-9.113936809	1.20E-17	1.03E-15	29.42023588
MS4A6A	-1.008021006	8.866574149	-9.100849621	1.32E-17	1.13E-15	29.32768215
SLCO2B1	-0.827975939	9.000382232	-9.100366879	1.33E-17	1.13E-15	29.32426959
PPM1M	-0.507466961	8.01177853	-9.092070257	1.41E-17	1.20E-15	29.2656355

EGR2	-0.889327505	7.548780596	-9.063198269	1.73E-17	1.46E-15	29.06182488
STK4	-0.500437488	9.625826204	-9.058429406	1.79E-17	1.51E-15	29.02819604
LPAR6	-0.686178139	7.998246845	-9.055567184	1.83E-17	1.53E-15	29.00801715
PLEK	-0.935281433	8.576746861	-9.05057965	1.90E-17	1.57E-15	28.97286323
DGKA	-0.622207075	6.829936523	-9.050420945	1.90E-17	1.57E-15	28.9717448
FCN1	-0.981437803	7.33137458	-9.035763403	2.11E-17	1.73E-15	28.86849752
CD84	-0.627696664	6.828346145	-9.031622598	2.18E-17	1.77E-15	28.83934695
LYZ	-1.561780057	9.488291116	-9.029976197	2.20E-17	1.78E-15	28.82775865
NKG7	-1.188271193	8.076540926	-9.019301877	2.38E-17	1.92E-15	28.75265584
LYVE1	-0.770303454	6.097968792	-9.003714153	2.66E-17	2.14E-15	28.64307356
F13A1	-1.142534614	7.191735664	-8.992760127	2.87E-17	2.30E-15	28.56613073
SLFN11	-0.674719696	8.424039221	-8.988963856	2.95E-17	2.35E-15	28.53947754
CCND2	-0.932330772	9.303249812	-8.978928935	3.17E-17	2.52E-15	28.46905432
APOBEC3G	-0.859162422	7.32540729	-8.975856258	3.24E-17	2.57E-15	28.44749979
FUCA1	-0.756404063	9.119054015	-8.955980193	3.74E-17	2.95E-15	28.30817283
HLA-E	-0.734696536	9.569034885	-8.951122763	3.87E-17	3.04E-15	28.27415011
EVL	-0.685843587	10.82404809	-8.922664529	4.74E-17	3.72E-15	28.07503342
CD34	-0.714191921	8.643811076	-8.919565481	4.85E-17	3.79E-15	28.05337193
CYBB	-0.798738442	7.571410301	-8.901030841	5.53E-17	4.29E-15	27.92391021
LTA	-0.671218753	6.582587084	-8.864102971	7.19E-17	5.51E-15	27.66643713
TMEM66	-0.509959042	11.10593027	-8.842951437	8.36E-17	6.38E-15	27.51924053
MAL	-0.996301057	6.841966959	-8.81956248	9.86E-17	7.49E-15	27.35671093
GAS7	-0.685945432	6.918667375	-8.8180733	9.97E-17	7.53E-15	27.3463711
EPAS1	-0.712171466	9.043359471	-8.817927032	9.98E-17	7.53E-15	27.34535558
SRGN	-1.121940793	9.87902144	-8.81400631	1.03E-16	7.69E-15	27.31813794
PTPN7	-0.671493009	6.610714599	-8.807391455	1.08E-16	8.04E-15	27.27223362
BCL11B	-0.859527466	6.998392195	-8.801150775	1.12E-16	8.35E-15	27.22894433
ZFP36	-0.857532263	9.51545877	-8.794349894	1.18E-16	8.73E-15	27.18178951
CDH5	-0.635249879	7.472531708	-8.778343889	1.32E-16	9.75E-15	27.07089369
FABP4	-1.497801018	7.223273025	-8.748000119	1.64E-16	1.20E-14	26.8609849
RASSF2	-0.733278737	9.049772859	-8.744967793	1.67E-16	1.22E-14	26.84003163
LY86	-0.719651482	8.114564417	-8.731502326	1.84E-16	1.34E-14	26.74703729
TNFRSF1B	-0.558154729	7.030280171	-8.71064735	2.13E-16	1.54E-14	26.6031765
PRCP	-0.563943519	8.689282716	-8.710308272	2.13E-16	1.54E-14	26.60083917
STAMBPL1	-0.628208142	7.396418159	-8.702691786	2.25E-16	1.62E-14	26.54835126
CD79A	-1.490060977	7.831242743	-8.684061411	2.56E-16	1.84E-14	26.42007709
IL18RAP	-0.524585485	6.195250706	-8.681085811	2.62E-16	1.87E-14	26.39960449
TSHZ2	-0.72131846	7.355594706	-8.65730833	3.09E-16	2.20E-14	26.23616111
C3	-1.093984223	8.453095752	-8.652421924	3.20E-16	2.27E-14	26.20260554
DPT	-0.948429202	7.338802104	-8.591007206	4.92E-16	3.44E-14	25.7818258
C1orf54	-0.613644018	8.935821146	-8.573816734	5.54E-16	3.87E-14	25.66436731
CPA3	-1.04258375	6.554591227	-8.549610016	6.56E-16	4.55E-14	25.49920799
NCKAP1L	-0.543253134	6.806724935	-8.546506469	6.70E-16	4.64E-14	25.47805321
LOC388152	0.779072236	8.201484769	8.538514594	7.09E-16	4.89E-14	25.42359928
VPREB3	-0.915051374	6.485164169	-8.536214001	7.20E-16	4.96E-14	25.40792951
GIPC1	0.580684014	10.21842235	8.527109726	7.67E-16	5.25E-14	25.34594357
CSF1R	-0.707539512	10.20540493	-8.522292584	7.93E-16	5.40E-14	25.31316248
PLCB2	-0.564721228	6.937702732	-8.48421485	1.03E-15	6.99E-14	25.05443454
GPC3	-0.508528676	5.967543963	-8.475845008	1.09E-15	7.39E-14	24.99765789
GEM	-0.768082502	6.949338835	-8.474941399	1.10E-15	7.40E-14	24.99153031
PDK4	-0.897436664	6.793098749	-8.474708228	1.10E-15	7.40E-14	24.98994919
BTG1	-0.566544259	11.26481493	-8.474073607	1.11E-15	7.42E-14	24.98564599
TPSAB1	-1.229500267	7.27640929	-8.468670904	1.15E-15	7.68E-14	24.94901959
ADH1A	-1.34723601	6.780072974	-8.463420818	1.19E-15	7.92E-14	24.91344144
AP1S2	-0.617399208	8.337216478	-8.45898393	1.23E-15	8.14E-14	24.88338454
FOS	-1.291492292	9.219095171	-8.458288146	1.24E-15	8.16E-14	24.87867194
TRIP13	0.874134923	7.918441508	8.45514193	1.26E-15	8.32E-14	24.85736534
SLC2A1	0.985635448	9.176473266	8.424566431	1.56E-15	1.02E-13	24.65055574
SERPINF1	-0.798559117	8.208590512	-8.423088427	1.58E-15	1.03E-13	24.64057027
NDN	-0.883207158	8.438356518	-8.406650141	1.76E-15	1.15E-13	24.52958455
CHAF1B	0.661445041	7.285867671	8.400688159	1.84E-15	1.19E-13	24.48936409
CD74	-0.847440673	8.263901791	-8.399884515	1.85E-15	1.20E-13	24.48394392
CTSW	-0.513723335	6.141053867	-8.39162083	1.96E-15	1.26E-13	24.42822802

HSD17B11	-0.798803376	7.441819372	-8.384621093	2.05E-15	1.31E-13	24.38106031
STK17B	-0.506896928	6.848265065	-8.382891928	2.08E-15	1.33E-13	24.36941206
OLFML1	-0.580746409	6.752752758	-8.366854071	2.32E-15	1.47E-13	24.26144592
ENG	-0.562717625	8.053904143	-8.365043491	2.35E-15	1.48E-13	24.24926517
CXCR6	-0.573541871	6.251383218	-8.351788552	2.57E-15	1.61E-13	24.16014148
CD1B	-0.575096215	5.82312204	-8.349520128	2.61E-15	1.63E-13	24.14489776
6-Sep	-0.616160799	6.349573523	-8.329979503	2.99E-15	1.85E-13	24.01369141
CPXM1	-0.739870803	6.830005921	-8.328254391	3.02E-15	1.86E-13	24.0021172
TBXAS1	-0.659392833	7.478011386	-8.328185641	3.02E-15	1.86E-13	24.00165597
HLA-DMB	-0.797393102	11.08334343	-8.322843232	3.14E-15	1.93E-13	23.96582199
PTPN6	-0.625054658	8.593480067	-8.319774946	3.20E-15	1.96E-13	23.94524803
PNOC	-0.569977045	5.895074299	-8.313836198	3.34E-15	2.04E-13	23.90543992
PRDM1	-0.667657966	7.833310033	-8.302792594	3.60E-15	2.19E-13	23.83146023
PRC1	0.805118268	9.280623134	8.302705401	3.60E-15	2.19E-13	23.83087638
FGD2	-0.724628223	8.077789443	-8.2860543	4.04E-15	2.45E-13	23.7194489
TOX2	-0.774142242	7.531035532	-8.279691379	4.21E-15	2.55E-13	23.67690559
LOC90925	-0.507571659	5.870876447	-8.270221304	4.50E-15	2.71E-13	23.61362509
OAF	-0.66572549	8.892987061	-8.233692184	5.77E-15	3.45E-13	23.36995513
SERPING1	-0.720357754	9.385370164	-8.227323161	6.02E-15	3.59E-13	23.32753915
CIRBP	-0.584097167	10.27544071	-8.224743989	6.13E-15	3.64E-13	23.3103684
ZCCHC24	-0.699356835	7.72170742	-8.207089042	6.91E-15	4.09E-13	23.19292177
C1orf112	0.58277078	7.47631826	8.207054024	6.91E-15	4.09E-13	23.19268898
IGFLR1	-0.674427447	7.685561256	-8.206739347	6.92E-15	4.09E-13	23.19059709
FOSB	-1.254215125	7.945844117	-8.201897517	7.16E-15	4.21E-13	23.15841613
GMPS	0.60952317	9.15982858	8.191949242	7.66E-15	4.50E-13	23.09233291
CBX7	-0.541105336	7.135637261	-8.190488904	7.73E-15	4.53E-13	23.08263659
CETP	-0.573415995	6.477736839	-8.182704804	8.15E-15	4.75E-13	23.03097024
OGN	-0.797398495	5.950298361	-8.163873861	9.26E-15	5.39E-13	22.9061093
CD97	-0.681100656	8.681250278	-8.161594089	9.40E-15	5.46E-13	22.89100528
CXCL13	-1.175594941	7.283694733	-8.130668378	1.16E-14	6.65E-13	22.68637868
CD7	-0.801537814	7.064423802	-8.129756381	1.17E-14	6.67E-13	22.68035171
TCF4	-0.674361985	8.514270606	-8.114916529	1.29E-14	7.36E-13	22.58234221
HBA2	-1.373910949	8.740494535	-8.113576092	1.30E-14	7.41E-13	22.57349491
CDC42SE2	-0.534424568	7.914075874	-8.101522172	1.41E-14	8.01E-13	22.493977
SEPP1	-1.326980229	8.469887882	-8.095052307	1.47E-14	8.33E-13	22.45132723
HSPB6	-0.713388005	6.325344663	-8.083447905	1.59E-14	8.95E-13	22.37488456
TESC	-0.507629968	6.174154492	-8.049904699	2.00E-14	1.11E-12	22.15431562
HLA-DQA1	-1.15565606	10.5703988	-8.04892446	2.01E-14	1.12E-12	22.14787871
LAMA2	-0.543418426	6.284076295	-8.034474839	2.21E-14	1.23E-12	22.05305079
PPAP2B	-0.70799424	8.813978856	-8.026123755	2.34E-14	1.29E-12	21.99829523
TSC22D3	-0.77303138	8.137423635	-8.017207347	2.49E-14	1.36E-12	21.9398733
APLNR	-0.645537039	7.886796588	-8.01149634	2.58E-14	1.41E-12	21.90247562
TPX2	0.74458189	7.60670587	7.993261271	2.92E-14	1.59E-12	21.78318059
HBB	-1.448458445	8.479283345	-7.991190696	2.96E-14	1.61E-12	21.7696458
CD33	-0.528767368	6.587749174	-7.983245324	3.12E-14	1.69E-12	21.71773
FERMT3	-0.682152711	8.479508338	-7.983004809	3.12E-14	1.69E-12	21.71615897
CYFIP2	-0.830581758	8.596964222	-7.977771688	3.23E-14	1.74E-12	21.6819841
TRPV2	-0.586577051	7.281424907	-7.970104091	3.40E-14	1.83E-12	21.63193698
BHLHE22	-0.598630035	6.381633225	-7.966209984	3.49E-14	1.88E-12	21.60653166
FCER1A	-1.049720323	6.901157668	-7.9535212	3.80E-14	2.03E-12	21.52380513
TNFRSF4	-0.54497193	7.709676971	-7.943684919	4.06E-14	2.16E-12	21.45973458
DUSP1	-1.083933725	9.702176852	-7.941985595	4.11E-14	2.18E-12	21.44867088
HJURP	0.659669165	7.373756262	7.93437663	4.32E-14	2.29E-12	21.39915044
HLA-DPB1	-0.74178197	7.312299756	-7.922593125	4.67E-14	2.46E-12	21.32252206
C16orf59	0.51765644	6.923653036	7.896580848	5.55E-14	2.91E-12	21.15362539
GINS2	0.81755334	8.166998789	7.880345951	6.18E-14	3.23E-12	21.04839595
SLIT3	-0.641113238	6.577920078	-7.865840483	6.80E-14	3.55E-12	20.95449551
LSR	0.603655992	7.363648231	7.864416875	6.87E-14	3.57E-12	20.94528593
TLR7	-0.638754746	7.081912261	-7.847618669	7.67E-14	3.95E-12	20.83669756
SYT11	-0.61216805	8.420423913	-7.830868673	8.57E-14	4.39E-12	20.72857207
PPAP2A	-0.658491393	8.05509259	-7.825526907	8.88E-14	4.54E-12	20.69412143
APOBEC3F	-0.524531296	6.704826978	-7.824567161	8.94E-14	4.56E-12	20.68793337
ITPR1	-0.596521027	6.87635464	-7.821342497	9.13E-14	4.64E-12	20.66714569

ZNF165	0.556770164	6.958657748	7.81937763	9.25E-14	4.69E-12	20.654482
CD83	-0.559797474	8.353052924	-7.816715624	9.41E-14	4.77E-12	20.63732853
ANTXR2	-0.52617228	8.059874347	-7.805863058	1.01E-13	5.08E-12	20.56743633
SFRP4	-1.238437505	8.334361381	-7.801180504	1.04E-13	5.22E-12	20.53729965
STIL	0.626039214	8.054603981	7.786903793	1.14E-13	5.71E-12	20.44548889
PAQR4	0.655703929	8.912862421	7.784178359	1.17E-13	5.80E-12	20.42797474
IGFBP6	-0.69791353	7.281112317	-7.7836836	1.17E-13	5.80E-12	20.42479576
NR2F6	0.544125107	8.373229644	7.76755755	1.30E-13	6.41E-12	20.32125374
CDC20	0.988554301	10.10738649	7.765888223	1.31E-13	6.47E-12	20.31054342
IL18BP	-0.743727714	8.75958996	-7.76515126	1.32E-13	6.49E-12	20.30581559
KIF2C	0.680737338	7.605962538	7.758853683	1.38E-13	6.74E-12	20.26542688
ATP2A3	-0.567856553	6.258355241	-7.753709907	1.42E-13	6.95E-12	20.23245399
CASP4	-0.573161392	8.801714566	-7.752359068	1.44E-13	6.99E-12	20.22379718
CTSH	-0.60816089	10.98347544	-7.742787804	1.53E-13	7.39E-12	20.16248851
CEP70	0.508806264	6.807697071	7.732316731	1.64E-13	7.90E-12	20.09547355
C13orf15	-0.706238919	9.83328476	-7.730828658	1.65E-13	7.96E-12	20.08595474
SLIT2	-0.523303329	7.097530214	-7.728617291	1.68E-13	8.05E-12	20.07181145
ARHGEF6	-0.622933132	8.999671294	-7.728610356	1.68E-13	8.05E-12	20.0717671
ANKRD29	-0.518078615	6.189300203	-7.709951792	1.90E-13	8.99E-12	19.95253905
HLA-DMA	-0.724164576	11.31161534	-7.704596205	1.96E-13	9.26E-12	19.91835224
DHDH	0.625644444	6.549166052	7.704280836	1.97E-13	9.26E-12	19.91633961
SMAP2	-0.505035493	7.328239677	-7.704109817	1.97E-13	9.26E-12	19.91524821
PARM1	-0.663590453	7.402980114	-7.702278719	1.99E-13	9.36E-12	19.90356371
FMO1	-0.765927828	6.929931062	-7.68873111	2.18E-13	1.01E-11	19.8171718
C17orf53	0.55562381	7.295932933	7.686688532	2.21E-13	1.02E-11	19.80415523
ICAM2	-0.610845463	8.294360469	-7.68667185	2.21E-13	1.02E-11	19.80404893
ICOS	-0.628215595	6.79270777	-7.670447278	2.45E-13	1.13E-11	19.70073797
TACC1	-0.612609883	9.910024856	-7.667141304	2.51E-13	1.15E-11	19.67970483
PDGFRA	-0.622932216	8.054299148	-7.655657567	2.70E-13	1.24E-11	19.60669045
RNASEH2A	0.512410428	7.595941211	7.650248733	2.80E-13	1.28E-11	19.57232604
ITGB2	-0.746690088	11.00675936	-7.632660019	3.14E-13	1.43E-11	19.46069043
GZMB	-1.09055608	7.769769239	-7.625205444	3.29E-13	1.49E-11	19.41342814
MAFB	-0.620686439	9.167560091	-7.622164478	3.36E-13	1.52E-11	19.39415719
FAM43A	-0.505238446	7.324564687	-7.618492813	3.44E-13	1.55E-11	19.37089629
GPSM2	0.660443026	7.654197154	7.612475243	3.57E-13	1.61E-11	19.33278975
LGMN	-0.714559216	9.01760027	-7.58801274	4.19E-13	1.87E-11	19.17808813
VEGFA	0.780774816	7.596988532	7.575608086	4.54E-13	2.02E-11	19.09976873
CAV1	-0.756395819	8.715676373	-7.542059888	5.63E-13	2.49E-11	18.88838813
CD93	-0.641979525	9.018220795	-7.541116307	5.67E-13	2.50E-11	18.88245197
C18orf56	0.767958004	7.6347069	7.529896971	6.09E-13	2.67E-11	18.81190861
DUSP5	-0.627799697	7.965555525	-7.527513622	6.19E-13	2.70E-11	18.79693208
KIFC1	0.683372835	7.617537729	7.521946857	6.41E-13	2.79E-11	18.76196405
AIF1	-0.712671207	8.108936267	-7.52015619	6.48E-13	2.82E-11	18.75071957
FHL1	-0.753231713	6.939292826	-7.517247874	6.61E-13	2.86E-11	18.7324607
LCP1	-0.806557999	10.94648763	-7.50357097	7.21E-13	3.11E-11	18.64665915
AGBL5	0.549071171	7.842391189	7.49576512	7.58E-13	3.24E-11	18.59773699
CCDC80	-0.533160818	6.166305884	-7.488823814	7.93E-13	3.38E-11	18.55426231
TEAD4	0.559071151	7.309657757	7.484433268	8.16E-13	3.47E-11	18.52677766
TNFRSF17	-0.931989872	6.637045677	-7.476414269	8.59E-13	3.64E-11	18.47660734
WDR34	0.537586547	8.045088651	7.455218241	9.83E-13	4.16E-11	18.3441723
LTB	-1.188850535	8.805722407	-7.448145908	1.03E-12	4.34E-11	18.30004068
SPRY1	-0.722009614	8.662212356	-7.443812101	1.06E-12	4.46E-11	18.2730117
CCNDBP1	-0.590517309	9.364662595	-7.430245181	1.15E-12	4.84E-11	18.18846741
OVOL2	0.72452577	8.492821655	7.423423012	1.20E-12	5.04E-11	18.14599391
SP110	-0.550753709	8.309534869	-7.417316721	1.25E-12	5.23E-11	18.10799999
LIPA	-0.733191806	9.791160704	-7.416077279	1.26E-12	5.27E-11	18.10029068
CRISPLD2	-0.775195648	9.30258756	-7.414173072	1.28E-12	5.32E-11	18.08844826
PSRC1	0.561178546	7.292255654	7.413671324	1.28E-12	5.33E-11	18.0853282
C5orf39	-0.646653636	7.535179632	-7.380488629	1.58E-12	6.46E-11	17.87930707
RTKN	0.614779617	9.520357183	7.377926149	1.61E-12	6.56E-11	17.86342384
KIF23	0.541177836	6.967210448	7.373379672	1.66E-12	6.74E-11	17.83525238
C1orf106	0.936495556	8.404931063	7.366637777	1.73E-12	7.00E-11	17.7934994
TNFRSF14	-0.606793163	9.941740406	-7.363296994	1.77E-12	7.11E-11	17.77281946

NCF1	-0.62049997	6.531166449	-7.359024677	1.81E-12	7.28E-11	17.74638261
LOC338758	-0.530842922	7.424695177	-7.353394108	1.88E-12	7.51E-11	17.71155715
TMEM140	-0.523680446	8.021983677	-7.337633804	2.08E-12	8.26E-11	17.61417635
RGS5	-0.54960141	6.722172038	-7.33733958	2.08E-12	8.26E-11	17.61235975
MS4A7	-0.667440461	7.562069839	-7.324671054	2.25E-12	8.92E-11	17.53418948
LIME1	-0.694586563	8.487076405	-7.324417722	2.26E-12	8.92E-11	17.53262726
C11orf96	-0.680406168	8.326920589	-7.313677036	2.42E-12	9.50E-11	17.46642739
FLAD1	0.55616847	9.558456236	7.306869914	2.52E-12	9.90E-11	17.42450674
SCG2	-0.579403366	5.930565035	-7.302205966	2.60E-12	1.02E-10	17.39580013
SLC25A10	0.625578648	7.517745039	7.298183036	2.66E-12	1.04E-10	17.3710492
IQGAP2	-0.659375065	6.836742952	-7.291939503	2.77E-12	1.08E-10	17.33265483
KIF20A	0.661917797	7.860980201	7.286447908	2.87E-12	1.11E-10	17.29890333
KIAA1949	-0.516911514	8.896601287	-7.275004178	3.08E-12	1.20E-10	17.2286266
RFTN1	-0.615636505	9.447019176	-7.271064946	3.16E-12	1.22E-10	17.20445328
CPZ	-0.532645105	6.338158655	-7.262608387	3.33E-12	1.29E-10	17.15258989
POU2AF1	-0.741413162	6.418622539	-7.258818047	3.41E-12	1.32E-10	17.12935767
CD248	-0.790596229	8.602386723	-7.258640706	3.42E-12	1.32E-10	17.1282709
SUSD3	-0.579902137	7.02850735	-7.244430158	3.74E-12	1.43E-10	17.04124661
FKBP4	0.616899593	8.208648204	7.244026674	3.74E-12	1.43E-10	17.03877744
CXCL14	-0.94500573	7.59069525	-7.242623422	3.78E-12	1.44E-10	17.03019082
PIM2	-0.883133883	8.255194694	-7.241392204	3.81E-12	1.45E-10	17.02265785
CKAP2L	0.53970586	7.136805999	7.232644205	4.02E-12	1.53E-10	16.96916061
SYK	-0.58418575	8.307942451	-7.220391889	4.34E-12	1.64E-10	16.89430912
CASP1	-0.595300579	8.423846617	-7.214444834	4.51E-12	1.70E-10	16.85800954
IGSF6	-0.63101939	7.330004107	-7.196987341	5.03E-12	1.89E-10	16.7515736
AURKA	0.671848479	8.195507102	7.184181537	5.44E-12	2.04E-10	16.6736133
HLA-DRA	-0.819800413	12.50873578	-7.182609165	5.50E-12	2.05E-10	16.66404759
CIDEC	-1.033268619	6.430257362	-7.180197687	5.58E-12	2.08E-10	16.64937993
SLC6A8	0.52979802	6.805468986	7.172839925	5.84E-12	2.17E-10	16.60464821
CDO1	-0.521785373	6.015734321	-7.166113587	6.09E-12	2.26E-10	16.56378344
CILP	-1.119938393	8.528471634	-7.162350041	6.24E-12	2.31E-10	16.5409304
LBH	-0.586409214	8.155317003	-7.161858586	6.26E-12	2.31E-10	16.53794679
CENPF	0.747100761	7.838749969	7.158165511	6.40E-12	2.36E-10	16.51553096
PODN	-0.901588198	8.354359512	-7.154849945	6.54E-12	2.41E-10	16.49541341
FOXM1	0.737675923	7.224180774	7.146718969	6.87E-12	2.51E-10	16.44610563
HDC	-0.578934827	6.023366331	-7.142988731	7.04E-12	2.56E-10	16.42349798
LST1	-0.616547298	7.512151577	-7.140689351	7.14E-12	2.60E-10	16.40956639
MYH11	-1.129621559	8.210512845	-7.136486599	7.33E-12	2.66E-10	16.38411074
SEL1L3	-0.696576742	7.971238411	-7.130814385	7.59E-12	2.74E-10	16.34977147
JAM3	-0.617499468	8.170044854	-7.128669008	7.69E-12	2.78E-10	16.33678849
SOCS2	-0.718445789	7.521299975	-7.121429521	8.04E-12	2.89E-10	16.29299833
STEAP3	0.643500588	8.563168098	7.102843186	9.02E-12	3.23E-10	16.18071742
SGK1	-0.596042105	9.645058314	-7.090018312	9.77E-12	3.49E-10	16.10336283
DAB2	-0.615191949	9.663413504	-7.075310635	1.07E-11	3.81E-10	16.01477386
MNDA	-0.515890135	6.50714151	-7.05737163	1.20E-11	4.25E-10	15.90689835
CTSC	-0.715819628	9.225510255	-7.052834641	1.23E-11	4.36E-10	15.87964617
TYROBP	-0.736970363	10.67127421	-7.051136869	1.24E-11	4.40E-10	15.86945143
CTSK	-0.909253598	9.891716109	-7.043235874	1.30E-11	4.60E-10	15.82203067
ELF3	0.836078025	8.847544287	7.041426857	1.32E-11	4.64E-10	15.81117851
UBQLN4	0.549523269	10.01494098	7.039845294	1.33E-11	4.68E-10	15.80169245
DCLK1	-0.621278511	7.013537467	-7.034002327	1.38E-11	4.85E-10	15.76666007
KRTCAP3	0.618839949	7.077048418	7.023851493	1.47E-11	5.14E-10	15.70584852
IRF6	0.683904682	7.823692666	7.01288667	1.57E-11	5.48E-10	15.64023091
TMEM54	0.599481749	8.773320177	7.009191427	1.61E-11	5.59E-10	15.61813368
CTSO	-0.562635106	7.709099937	-7.005231411	1.65E-11	5.71E-10	15.59446236
HMGB3	0.657230547	7.678782976	7.000185392	1.70E-11	5.87E-10	15.56431321
LIMA1	-0.510960491	7.673893059	-6.991753089	1.79E-11	6.16E-10	15.51396623
ECE2	0.563249233	7.948095852	6.977345042	1.95E-11	6.66E-10	15.42804022
SRPX	-0.860601909	8.170572393	-6.957084173	2.21E-11	7.51E-10	15.30742437
MARVELD3	0.561307098	7.353397514	6.956020882	2.23E-11	7.55E-10	15.3011014
CCNE1	0.805075113	7.609716995	6.953938147	2.25E-11	7.64E-10	15.2887182
CENPA	0.647574889	7.595149179	6.947022319	2.35E-11	7.91E-10	15.24761827
C1orf135	0.579637008	6.892891902	6.941463141	2.43E-11	8.17E-10	15.21460206

CR2	-0.622417368	5.896255378	-6.909827791	2.95E-11	9.81E-10	15.0270804
FGR	-0.615565975	7.820152439	-6.901039763	3.11E-11	1.03E-09	14.97509807
STAB1	-0.564658787	7.315865861	-6.898715779	3.15E-11	1.04E-09	14.96135939
ID3	-0.750420672	8.846416047	-6.893810515	3.25E-11	1.07E-09	14.93237192
MATK	-0.657371664	7.009620275	-6.889248549	3.34E-11	1.10E-09	14.90542654
STX11	-0.518604879	6.972637316	-6.882638415	3.48E-11	1.14E-09	14.86640645
CDCA5	0.686648172	9.292436642	6.87347898	3.67E-11	1.20E-09	14.81238243
NCF1C	-0.524103255	6.675885949	-6.856108796	4.08E-11	1.33E-09	14.71007297
FBLN1	-0.782757299	10.3439319	-6.85351585	4.15E-11	1.35E-09	14.69481674
C1QA	-0.668457895	7.846301896	-6.847537645	4.30E-11	1.40E-09	14.65965848
RHOD	0.524579851	8.020585092	6.82662043	4.88E-11	1.57E-09	14.53681809
EFEMP1	-1.010187556	8.536978466	-6.823019896	4.98E-11	1.60E-09	14.5157008
IFI16	-0.628922439	8.53165055	-6.819125928	5.10E-11	1.63E-09	14.49287164
KLF9	-0.591738142	8.779984115	-6.809649275	5.40E-11	1.72E-09	14.43735248
GPR68	-0.523219584	6.934003297	-6.807740057	5.46E-11	1.74E-09	14.42617409
GPX3	-0.755727058	8.442835596	-6.803834743	5.59E-11	1.78E-09	14.40331575
PTPRF	0.661917332	9.488770949	6.798181266	5.79E-11	1.83E-09	14.37024211
FAM113B	-0.656966143	8.182279484	-6.798072245	5.79E-11	1.83E-09	14.36960452
TGFB3	-0.651776143	7.907515872	-6.791548315	6.02E-11	1.90E-09	14.33146395
CDCP1	0.527396682	7.264608132	6.782774021	6.35E-11	1.99E-09	14.28020931
EFNA1	0.697268066	10.45044097	6.774170252	6.68E-11	2.09E-09	14.22999775
UBE2C	0.895723956	10.04084584	6.773209354	6.72E-11	2.10E-09	14.22439284
SKAP1	-0.688401248	7.400262357	-6.754415163	7.52E-11	2.34E-09	14.11488341
PLTP	-0.591022597	9.429523764	-6.751458995	7.66E-11	2.38E-09	14.09767876
PKIA	-0.570147398	6.665513184	-6.748855048	7.78E-11	2.41E-09	14.08252858
TBC1D9	-0.617524076	7.417714878	-6.740308372	8.18E-11	2.53E-09	14.03283271
CHN1	-0.582424519	8.157861231	-6.739667128	8.21E-11	2.53E-09	14.02910597
UBE2S	0.631090822	9.781708574	6.725700768	8.93E-11	2.73E-09	13.94800162
MOCOS	0.795190558	7.681173695	6.725451083	8.94E-11	2.73E-09	13.94655278
CDC45	0.620438843	8.033725302	6.724169222	9.01E-11	2.74E-09	13.93911525
ASF1B	0.526002633	7.023509529	6.723781872	9.03E-11	2.75E-09	13.936868
IGSF3	0.650371523	8.542291834	6.713632166	9.59E-11	2.91E-09	13.87801723
SPOCK2	-0.977846161	8.937882665	-6.699354997	1.04E-10	3.15E-09	13.79534489
ECM2	-0.668973043	7.404335459	-6.692152309	1.09E-10	3.27E-09	13.75368667
GPR34	-0.606286165	6.525486599	-6.687856244	1.12E-10	3.35E-09	13.7288552
PPAP2C	0.585246258	7.57258677	6.686320882	1.13E-10	3.37E-09	13.71998358
COLEC12	-0.792886168	7.612054594	-6.683632444	1.15E-10	3.42E-09	13.70445287
CDCA3	0.68677329	7.437138732	6.682985144	1.15E-10	3.43E-09	13.7007142
FKBP11	-0.654950545	9.373428248	-6.676258514	1.20E-10	3.55E-09	13.6618784
abParts	-1.757446371	11.51548287	-6.676098082	1.20E-10	3.55E-09	13.66095251
C1S	-0.716934573	10.28407889	-6.664595709	1.28E-10	3.78E-09	13.59461224
TTK	0.704453298	7.653624276	6.662985001	1.30E-10	3.81E-09	13.58532918
IL6	-0.634479433	6.427844701	-6.655816057	1.35E-10	3.96E-09	13.54403217
ASPM	0.685673856	8.18446227	6.643756352	1.45E-10	4.22E-09	13.47463584
EMILIN1	-0.50372273	7.074719531	-6.603737214	1.84E-10	5.29E-09	13.24501759
CRB3	0.636700359	8.135163404	6.602111477	1.86E-10	5.34E-09	13.23571133
RPL13A	-0.557490238	10.11280472	-6.582108983	2.09E-10	5.95E-09	13.12134963
PLOD1	0.534005227	10.78138428	6.577469397	2.15E-10	6.10E-09	13.09486026
CDCA2	0.528743665	6.741938186	6.576625082	2.16E-10	6.13E-09	13.0900412
HOOK1	0.639807768	8.054206116	6.575145516	2.18E-10	6.17E-09	13.08159746
CDCA8	0.600512167	7.569462738	6.561834957	2.35E-10	6.63E-09	13.00569893
ALPL	-0.864267197	8.318915445	-6.559681305	2.38E-10	6.70E-09	12.9934293
SLMO1	0.526570683	7.249983187	6.549226829	2.53E-10	7.07E-09	12.93391147
MZB1	-1.178028387	8.269890028	-6.547530591	2.56E-10	7.13E-09	12.92426138
UCK2	0.533562537	8.912575774	6.545849238	2.58E-10	7.18E-09	12.91469781
FAM46C	-1.069234505	8.578277614	-6.544345476	2.61E-10	7.23E-09	12.90614594
EGFLAM	-0.559740893	7.540074299	-6.540566906	2.66E-10	7.37E-09	12.88466374
ADCY4	-0.538560703	6.87629038	-6.538327394	2.70E-10	7.45E-09	12.87193588
GAPDH	0.547503249	10.92102532	6.534853539	2.75E-10	7.57E-09	12.85219928
ICAM3	-0.564169809	8.549886461	-6.534402572	2.76E-10	7.58E-09	12.8496377
CCNB2	0.683711433	8.918745439	6.515474949	3.08E-10	8.43E-09	12.7422443
COL15A1	-0.634086773	8.645763548	-6.509347427	3.20E-10	8.70E-09	12.7075273
RHPN2	0.834353956	9.230192549	6.505954474	3.26E-10	8.85E-09	12.6883142

GYPC	-0.709453243	8.348995074	-6.504787779	3.28E-10	8.90E-09	12.68170935
SPINT2	0.637035661	11.63097151	6.495480257	3.47E-10	9.35E-09	12.62904972
BUB1	0.588462034	7.390906885	6.495062767	3.47E-10	9.36E-09	12.626689
ECT2	0.563208799	8.060499625	6.490001857	3.58E-10	9.63E-09	12.59808074
CYBA	-0.579431379	11.04514802	-6.48034168	3.78E-10	1.01E-08	12.54352023
EXO1	0.596782237	7.573886092	6.479574014	3.80E-10	1.02E-08	12.53918708
SNCA	-0.504331343	6.67257827	-6.474019293	3.93E-10	1.05E-08	12.50784453
CGN	0.792553398	8.320557099	6.470163394	4.01E-10	1.07E-08	12.48609946
IGFBP7	-0.622424378	11.49096464	-6.469035601	4.04E-10	1.07E-08	12.47974119
PACSIN3	0.516821847	7.321098203	6.468739758	4.05E-10	1.07E-08	12.47807343
RAB25	0.816315568	9.693275064	6.467621776	4.07E-10	1.08E-08	12.47177151
CELSR2	0.525346322	6.601641148	6.461593446	4.22E-10	1.11E-08	12.43780471
EGR1	-0.78550018	9.051244901	-6.460775794	4.24E-10	1.11E-08	12.43319946
CBLC	0.552014658	6.570984917	6.44636596	4.61E-10	1.20E-08	12.35211111
ITGAX	-0.555089153	7.587048082	-6.445810367	4.62E-10	1.20E-08	12.34898735
CD36	-1.039073325	7.658688319	-6.43017422	5.06E-10	1.31E-08	12.26115814
CEP55	0.587025425	7.871367039	6.424013777	5.24E-10	1.36E-08	12.2265987
LAP3	-0.530631531	9.557397729	-6.415212526	5.52E-10	1.42E-08	12.17726797
EPB41L3	-0.554154634	7.584781179	-6.387784079	6.46E-10	1.64E-08	12.02386024
SEMA4D	-0.511433052	8.789242849	-6.366466858	7.30E-10	1.83E-08	11.9049763
CD38	-0.771233977	6.773312505	-6.363649009	7.42E-10	1.86E-08	11.88928399
SOX18	-0.580769891	8.747217777	-6.361765204	7.50E-10	1.88E-08	11.87879622
CPE	-0.694198881	7.420362093	-6.328199626	9.09E-10	2.25E-08	11.69232071
WISP2	-0.789262019	6.831113534	-6.325505349	9.23E-10	2.28E-08	11.67738502
TFAP2C	0.753241925	8.516574103	6.322514513	9.39E-10	2.31E-08	11.66081102
GLIPR1	-0.591844919	7.912366051	-6.310693818	1.00E-09	2.46E-08	11.59536395
TROAP	0.507396824	7.272141348	6.306471126	1.03E-09	2.51E-08	11.57200699
FILIP1L	-0.540666726	7.745306631	-6.303326692	1.05E-09	2.55E-08	11.55462194
CLDN11	-0.619802707	6.61236013	-6.302847759	1.05E-09	2.56E-08	11.55197458
CA9	0.872627903	6.605360032	6.299274134	1.07E-09	2.60E-08	11.53222577
MCM2	0.56078369	8.186398149	6.290610531	1.13E-09	2.72E-08	11.48438386
SH3BGRL	-0.5465667	9.260821987	-6.29020602	1.13E-09	2.72E-08	11.4821513
DDR1	0.574307722	8.804128393	6.280988216	1.19E-09	2.86E-08	11.43130668
TK1	0.610382281	8.677872725	6.276895489	1.22E-09	2.92E-08	11.40874981
DKFZp686O16217	-1.673454438	9.191222824	-6.276404337	1.22E-09	2.92E-08	11.4060436
HUAT	-0.515338163	7.47373083	-6.263931028	1.31E-09	3.11E-08	11.33737099
SLC2A3	-0.578536048	9.532898854	-6.263854031	1.31E-09	3.11E-08	11.3369474
GTPBP4	0.5251909	10.08971546	6.257896291	1.36E-09	3.20E-08	11.30418386
MCM4	0.619475779	9.104906075	6.257751796	1.36E-09	3.20E-08	11.30338953
FAM83H	0.626886299	8.79134955	6.255546275	1.37E-09	3.24E-08	11.29126693
TMEM176A	-0.530011905	7.407500948	-6.25504345	1.38E-09	3.24E-08	11.28850362
DBNDD1	0.70844261	8.546535374	6.252573766	1.40E-09	3.29E-08	11.27493376
SRD5A1	0.625458939	7.364365811	6.24557925	1.45E-09	3.41E-08	11.23652418
EVPL	0.550660374	7.322032937	6.244513605	1.46E-09	3.42E-08	11.23067519
SNRPN	-0.694924001	7.881689077	-6.236627608	1.53E-09	3.57E-08	11.18741532
CD163	-0.775497945	8.960062234	-6.228290679	1.60E-09	3.73E-08	11.14172737
ACACB	-0.530794464	7.267315519	-6.227165403	1.61E-09	3.75E-08	11.13556423
MELK	0.697178069	8.470357441	6.223532218	1.65E-09	3.83E-08	11.11567113
BIRC5	0.743378791	8.05248189	6.221041018	1.67E-09	3.87E-08	11.10203599
GPT2	0.653569275	8.906223237	6.217875135	1.70E-09	3.94E-08	11.08471414
NEK2	0.657830093	7.259388837	6.214472049	1.73E-09	4.01E-08	11.066102
CYGB	-0.509104469	7.687404421	-6.205582325	1.82E-09	4.20E-08	11.01751933
CMTM4	0.521378855	8.517689292	6.203942801	1.84E-09	4.24E-08	11.00856512
ETV4	0.522476192	6.278058194	6.180465927	2.10E-09	4.80E-08	10.88054651
RCOR2	0.624494545	6.902522413	6.172020498	2.20E-09	5.01E-08	10.83458532
NFS1	-0.687458541	8.061205541	-6.170538596	2.22E-09	5.05E-08	10.8265256
GPRIN2	0.652706672	6.496327655	6.165169069	2.29E-09	5.18E-08	10.79733451
PPARG	-0.639421794	7.48516922	-6.16416688	2.30E-09	5.20E-08	10.79188834
ITGB7	-0.580593358	7.486565964	-6.16163878	2.33E-09	5.27E-08	10.778153
SOX9	0.698709197	8.348028103	6.120055821	2.94E-09	6.52E-08	10.55285495
ZSWIM4	0.55118992	9.049008999	6.107036359	3.16E-09	6.97E-08	10.48255781
DSG2	0.543377066	7.200465942	6.101093986	3.27E-09	7.18E-08	10.45051121
CAMK1G	-0.809990072	6.831829438	-6.099412356	3.30E-09	7.24E-08	10.44144675

C2	-0.523734275	7.459006737	-6.095420315	3.37E-09	7.39E-08	10.41993628
LFNG	-0.647087994	8.399509888	-6.090445452	3.47E-09	7.59E-08	10.39314533
PLA2G2A	-0.831134206	6.243775713	-6.087540203	3.52E-09	7.70E-08	10.37750766
CDK1	0.586661729	7.862676583	6.086659859	3.54E-09	7.73E-08	10.3727703
PCDH18	-0.618754097	7.838640085	-6.08452705	3.58E-09	7.80E-08	10.36129531
COX7A1	-0.624518384	8.194926256	-6.083420775	3.61E-09	7.85E-08	10.35534453
NPL	-0.516646262	7.695386133	-6.080120026	3.67E-09	7.98E-08	10.33759442
FAM64A	0.513283399	7.006573073	6.078090771	3.71E-09	8.06E-08	10.32668562
C1QC	-0.63560971	10.46522781	-6.068391822	3.92E-09	8.47E-08	10.27458543
TXNDC5	-0.639405575	8.419280726	-6.066247972	3.97E-09	8.56E-08	10.26307797
TACC2	0.50108032	7.070447217	6.057181772	4.17E-09	8.97E-08	10.21444867
MCM10	0.628562576	7.617865762	6.049076102	4.36E-09	9.35E-08	10.17101941
LAPTM5	-0.564490718	9.255372784	-6.044275722	4.48E-09	9.57E-08	10.14532088
CABYR	0.565606208	7.165087998	6.025866861	4.96E-09	1.05E-07	10.04691776
TRIB3	0.592603057	8.012904689	6.019207966	5.14E-09	1.09E-07	10.01138086
MCOLN2	-0.537744386	7.730281443	-6.015780155	5.24E-09	1.10E-07	9.993099428
PLIN1	-0.501095585	5.825377536	-6.010535406	5.39E-09	1.13E-07	9.965143538
BIN1	-0.543441004	8.35323903	-6.010028522	5.41E-09	1.13E-07	9.962442728
HERPUD1	-0.620010311	9.458686933	-6.008641944	5.45E-09	1.14E-07	9.955055576
C1R	-0.729690243	7.599140693	-6.007335031	5.49E-09	1.15E-07	9.948094065
C1QB	-0.703795752	11.0309949	-5.99807611	5.78E-09	1.20E-07	9.898808677
BNIP3	0.565390315	9.565645248	5.997831842	5.78E-09	1.20E-07	9.897509238
COL6A3	-0.758947703	10.40534514	-5.986996453	6.14E-09	1.28E-07	9.839909675
TTLL4	0.568738273	7.31712974	5.986276092	6.16E-09	1.28E-07	9.836083215
TM4SF1	0.749350284	12.15643904	5.985230383	6.20E-09	1.28E-07	9.8305292
SLC6A10P	0.629106294	7.918380198	5.959183502	7.15E-09	1.46E-07	9.692433275
ITM2C	-0.602977341	9.42350544	-5.958792182	7.16E-09	1.46E-07	9.690362167
CD86	-0.511480406	8.224211861	-5.942241314	7.84E-09	1.59E-07	9.602862615
SPON1	-0.781879133	7.706549763	-5.939636672	7.95E-09	1.61E-07	9.589110061
RASD1	-0.745545913	7.723496549	-5.939361225	7.97E-09	1.61E-07	9.587655974
HLA-DOB	-0.595795187	7.07320967	-5.927333438	8.51E-09	1.71E-07	9.524212882
NUSAP1	0.523948317	8.714111853	5.925502744	8.59E-09	1.72E-07	9.514565376
CCL5	-0.894866273	10.72604341	-5.923784702	8.67E-09	1.74E-07	9.505513666
CX3CR1	-0.562439658	6.799646188	-5.910606079	9.32E-09	1.86E-07	9.436149254
PDGFRB	-0.572438234	9.827399175	-5.910361311	9.33E-09	1.86E-07	9.43486209
CCL22	-0.508621644	6.179297081	-5.864555906	1.20E-08	2.33E-07	9.19472611
SLC7A7	-0.536264234	8.859185418	-5.839710581	1.37E-08	2.64E-07	9.065092119
GZMH	-0.564653637	6.777808707	-5.837545512	1.38E-08	2.67E-07	9.053816226
ALOX5AP	-0.695177448	9.807678453	-5.835716189	1.40E-08	2.69E-07	9.044291507
PLIN4	-0.894430163	6.872509198	-5.816001356	1.55E-08	2.97E-07	8.941792842
CXADR	0.748895302	8.461979648	5.806174118	1.64E-08	3.10E-07	8.890803281
PCOLCE	-0.673655764	8.855212673	-5.805544907	1.64E-08	3.11E-07	8.887540895
ATAD2	0.517228861	7.654488439	5.802670935	1.67E-08	3.14E-07	8.872643252
TMEM125	0.585735914	7.633315593	5.801743574	1.68E-08	3.15E-07	8.867837397
GBP2	-0.625145276	10.51067751	-5.793376639	1.75E-08	3.28E-07	8.824505114
LAD1	0.842240001	10.14923604	5.787175367	1.81E-08	3.39E-07	8.792420884
RARRES2	-0.668580515	9.484710457	-5.757406627	2.12E-08	3.93E-07	8.638784143
IGKC	-0.652242898	6.016339211	-5.751582052	2.19E-08	4.05E-07	8.608797375
TMEM79	0.687218996	8.676461246	5.7314277	2.44E-08	4.47E-07	8.505223447
CD14	-0.564270865	9.742490432	-5.717832636	2.62E-08	4.78E-07	8.435522126
LILRB4	-0.512835662	7.19070132	-5.713902412	2.68E-08	4.86E-07	8.415396711
ADAMDEC1	-0.788274184	7.735521917	-5.699211783	2.89E-08	5.22E-07	8.340268888
C18orf45	0.52234382	7.377072847	5.698467028	2.90E-08	5.23E-07	8.336464343
FEZ1	-0.506387015	6.939750219	-5.692976708	2.99E-08	5.37E-07	8.308429598
CYBRD1	-0.56700287	8.865234635	-5.674561055	3.30E-08	5.89E-07	8.214553689
PRG4	-0.576160364	6.249180541	-5.658386843	3.59E-08	6.37E-07	8.132305316
GNLY	-0.829947248	7.844195297	-5.645651198	3.84E-08	6.75E-07	8.067675456
TOP2A	0.646441542	9.25928281	5.635582555	4.04E-08	7.08E-07	8.016662871
UBE2T	0.581745068	8.063894001	5.625698849	4.26E-08	7.43E-07	7.966658622
SFN	0.753816199	7.659068904	5.612400501	4.57E-08	7.93E-07	7.899490486
HOMER2	0.62064275	7.621314181	5.602622471	4.81E-08	8.29E-07	7.850184846
IGSF9	0.536692404	7.055710973	5.592555708	5.06E-08	8.69E-07	7.799495823
HOPX	-0.686514093	8.101942242	-5.579260817	5.43E-08	9.28E-07	7.732665197

TGM2	-0.524362232	8.204970337	-5.572189574	5.63E-08	9.58E-07	7.697171963
NLGN4X	-0.566873094	6.53576116	-5.559855847	6.00E-08	1.01E-06	7.635351581
STC2	0.655832202	7.95436857	5.559050402	6.03E-08	1.02E-06	7.631318303
HMGA1	0.564664141	9.206661751	5.557585282	6.07E-08	1.02E-06	7.623982914
ALOX5	-0.51532817	9.217054867	-5.545900124	6.45E-08	1.08E-06	7.565535139
THY1	-0.5606995	9.884628138	-5.545140447	6.48E-08	1.08E-06	7.56173878
SERTAD4	0.64419205	8.453868968	5.528318345	7.07E-08	1.18E-06	7.477781265
ETS1	-0.567464376	8.284442056	-5.521267331	7.33E-08	1.21E-06	7.44265194
CTSL2	0.674877552	7.36286242	5.516719355	7.51E-08	1.24E-06	7.420012495
UCP2	-0.637162335	8.770958067	-5.511603436	7.71E-08	1.27E-06	7.394564006
CDH11	-0.601518635	8.486375965	-5.50467723	7.99E-08	1.31E-06	7.360141071
PDGFRL	-0.652274169	8.381013352	-5.49958904	8.20E-08	1.34E-06	7.334875436
MLLT4	0.542214284	8.697282681	5.498265375	8.25E-08	1.35E-06	7.328305832
UBE2L6	-0.506230482	7.8657567	-5.496362772	8.34E-08	1.36E-06	7.318865107
TFAP2A	0.675553948	9.695395543	5.488636272	8.67E-08	1.41E-06	7.280553521
PRSS8	0.702558849	9.160616684	5.485183706	8.83E-08	1.43E-06	7.263448282
FAM198B	-0.54454999	7.901563784	-5.479874933	9.07E-08	1.46E-06	7.237163851
AURKB	0.55777351	7.869976116	5.475569031	9.28E-08	1.49E-06	7.215860003
VCAM1	-0.673595344	9.519287412	-5.467543338	9.66E-08	1.55E-06	7.176188598
CDK12	-0.616126411	7.734742799	-5.465259668	9.78E-08	1.57E-06	7.16490898
CD24	0.756085089	13.23152069	5.459307285	1.01E-07	1.61E-06	7.135526749
ASNS	0.518161125	9.207284728	5.454141563	1.04E-07	1.65E-06	7.110048828
CLDN3	0.806555516	8.224083875	5.451575415	1.05E-07	1.67E-06	7.097399623
CRTAP	-0.561358151	7.910547232	-5.449937951	1.06E-07	1.68E-06	7.089330682
NR2F1	-0.561844474	6.828165372	-5.402567346	1.35E-07	2.10E-06	6.856761071
EIF2C2	0.532230363	9.575226618	5.391588698	1.42E-07	2.20E-06	6.803098047
DSC2	0.59476706	7.300833299	5.38951407	1.44E-07	2.23E-06	6.792967438
3-Sep	0.752850541	7.644005708	5.38035867	1.51E-07	2.32E-06	6.748298978
DSP	0.678462977	9.091451885	5.375844516	1.54E-07	2.36E-06	6.72629774
SFRP2	-0.914758036	9.994249041	-5.375452386	1.54E-07	2.37E-06	6.72438728
KLF4	-0.51952479	6.991614953	-5.37232331	1.57E-07	2.40E-06	6.709146497
HTRA1	-0.673060679	10.72155266	-5.368994065	1.60E-07	2.43E-06	6.692938763
TGFBR3	-0.650694513	8.230599306	-5.366225998	1.62E-07	2.46E-06	6.679469294
ISG20	-0.606919458	9.672059456	-5.35778651	1.69E-07	2.56E-06	6.638437829
F12	0.629649334	7.860358505	5.357518969	1.69E-07	2.56E-06	6.637137953
XBP1	-0.635706222	11.1885613	-5.354179957	1.72E-07	2.60E-06	6.620919536
IMPA2	0.623907657	8.867215179	5.349152699	1.76E-07	2.66E-06	6.596516579
KRT7	0.819723626	10.02456557	5.337157957	1.87E-07	2.81E-06	6.538368802
GALNTL1	-0.594158532	6.930517476	-5.322362636	2.02E-07	3.00E-06	6.466792638
ZNF683	-0.519223675	6.730846063	-5.285771609	2.42E-07	3.56E-06	6.290478662
PLA2G7	-0.600587096	7.741580592	-5.281735758	2.47E-07	3.62E-06	6.271093458
FCER1G	-0.583067642	9.906712646	-5.231746537	3.17E-07	4.54E-06	6.03199979
LUM	-0.810286233	10.99635643	-5.199293185	3.72E-07	5.25E-06	5.877789188
RGS1	-0.656014299	8.842944703	-5.181917387	4.06E-07	5.67E-06	5.79555173
LRRC17	-0.600735551	6.730166653	-5.179342471	4.11E-07	5.74E-06	5.783384487
CCL4	-0.545254899	7.861737848	-5.17908471	4.11E-07	5.74E-06	5.782166765
TPT1	-0.522373544	10.66847728	-5.16452542	4.42E-07	6.13E-06	5.713467487
CHST3	0.506254865	7.803022189	5.158192928	4.56E-07	6.31E-06	5.68363741
THBS4	-0.765493214	7.380101211	-5.147055755	4.81E-07	6.65E-06	5.63124834
CCL2	-0.584315412	9.633548751	-5.138959008	5.01E-07	6.89E-06	5.593220753
LAMC2	0.725779079	7.30143039	5.120038692	5.49E-07	7.49E-06	5.504553795
DDIT4	0.578322767	10.04330026	5.118067204	5.54E-07	7.56E-06	5.495330481
C5orf46	0.862426367	8.111496224	5.115596679	5.61E-07	7.64E-06	5.483776691
KRT16P3	0.905015438	7.635747723	5.11432493	5.65E-07	7.66E-06	5.477830981
CCL13	-0.64410481	6.852684611	-5.10795731	5.82E-07	7.87E-06	5.448079559
CXCL9	-0.99993486	9.499397636	-5.076231059	6.79E-07	9.03E-06	5.300308103
PRAME	0.640086933	6.722609175	5.072571666	6.91E-07	9.17E-06	5.28331342
BC038245	0.514245313	7.16553885	5.055753448	7.50E-07	9.88E-06	5.205339877
ITGB4	0.635967218	7.830214819	5.055413395	7.51E-07	9.90E-06	5.20376555
CBX2	0.640253321	8.746202365	5.052859893	7.61E-07	9.99E-06	5.191946563
KREMEN2	0.519828011	6.410129409	5.038061885	8.17E-07	1.06E-05	5.123552262
RARRES3	-0.792986188	10.58668181	-5.036831748	8.22E-07	1.07E-05	5.11787434
PROS1	-0.538940131	7.931594909	-5.027303267	8.60E-07	1.11E-05	5.073933439

VGLL1	0.930921399	7.560738612	5.00557684	9.55E-07	1.23E-05	4.974003509
SQLE	0.522990448	8.974101105	5.000342964	9.79E-07	1.25E-05	4.949985032
RASD2	0.734006722	8.091335844	4.993868078	1.01E-06	1.29E-05	4.920300846
ERBB3	0.596467696	8.799066367	4.992056943	1.02E-06	1.30E-05	4.912003489
CXCR4	-0.69235411	8.899010514	-4.970914181	1.13E-06	1.42E-05	4.81533019
NELL2	-0.627333167	6.81796133	-4.963263402	1.17E-06	1.46E-05	4.780433188
HES6	0.513530124	7.579443859	4.953380527	1.22E-06	1.53E-05	4.735422376
APOD	-1.241692979	10.37683329	-4.94034141	1.30E-06	1.62E-05	4.676152918
KRT19	0.876623423	11.7728472	4.937532592	1.32E-06	1.63E-05	4.663402726
EPSTI1	-0.580655301	9.07857631	-4.919057561	1.44E-06	1.77E-05	4.579691412
ERAP2	-0.591773666	7.102019698	-4.914239872	1.47E-06	1.81E-05	4.557905973
VGF	0.537309213	6.579308706	4.905514305	1.54E-06	1.88E-05	4.51849536
IL17RB	0.585122601	7.365488166	4.90439434	1.54E-06	1.88E-05	4.513441142
CKS2	0.522018524	9.995148181	4.902978614	1.55E-06	1.89E-05	4.507053603
GCNT2	0.502631133	7.502964559	4.888904643	1.66E-06	2.00E-05	4.443639226
CYB5R2	0.565744804	8.211450347	4.864686076	1.86E-06	2.22E-05	4.334878393
C1orf116	0.707148573	7.903626245	4.859713544	1.90E-06	2.26E-05	4.31260462
CKS1B	0.586871636	10.51741722	4.846688888	2.02E-06	2.39E-05	4.254354467
MCF2L-AS1	0.523103988	7.415123508	4.836612543	2.12E-06	2.49E-05	4.209381487
ANGPTL2	-0.52352714	8.333448402	-4.830837583	2.18E-06	2.55E-05	4.183642541
FRZB	-0.652776816	7.519075555	-4.795569397	2.57E-06	2.96E-05	4.027022664
KRT8	0.651172232	9.454381105	4.786785923	2.67E-06	3.07E-05	3.98816942
SERINC2	0.524122223	7.422595626	4.783375841	2.72E-06	3.12E-05	3.973101528
PLOD2	0.539386866	10.08368869	4.768588185	2.91E-06	3.31E-05	3.907866737
DA738617	0.535470361	6.233030954	4.763339312	2.98E-06	3.39E-05	3.884753289
MT1G	0.795344686	9.091996368	4.757337491	3.06E-06	3.47E-05	3.858350974
CLDN7	0.591286321	8.546327055	4.750310086	3.16E-06	3.57E-05	3.827473341
PPP1R14C	0.66959843	7.008471032	4.713658928	3.74E-06	4.15E-05	3.667067627
IRF1	-0.506836646	9.458331663	-4.707146585	3.85E-06	4.26E-05	3.638677754
C10orf116	-0.742428209	9.75899627	-4.684936206	4.26E-06	4.67E-05	3.542108071
HIST1H2BD	0.593839355	8.993923835	4.67754847	4.41E-06	4.81E-05	3.51007374
MATN2	-0.53546955	7.380999762	-4.657213194	4.84E-06	5.22E-05	3.4221221
PITX1	0.781112683	9.307652725	4.646391036	5.08E-06	5.45E-05	3.37545027
IGLL3P	-0.875046626	7.505365613	-4.63021436	5.46E-06	5.81E-05	3.30586135
CIDEA	-0.571321733	6.215233969	-4.569295113	7.18E-06	7.40E-05	3.045684521
IGLL1	-0.922196323	12.51977797	-4.557415424	7.57E-06	7.75E-05	2.995296401
RAP1GAP	0.614391143	7.987822034	4.541577006	8.12E-06	8.26E-05	2.928294437
CDKN2A	0.661910928	7.211618423	4.529983271	8.55E-06	8.64E-05	2.879377501
LEMD1	0.60792544	6.296699072	4.503066619	9.62E-06	9.65E-05	2.766228941
TACSTD2	0.699146936	11.23094311	4.492259984	1.01E-05	0.000100843	2.720966782
TMEM158	0.784455428	9.361693849	4.472551692	1.10E-05	0.000108841	2.638665482
PROM2	0.622668574	8.856902003	4.465102534	1.14E-05	0.000112177	2.607640263
C8orf4	-0.572005226	7.125556032	-4.452184	1.20E-05	0.000117979	2.553942595
CITED4	0.61105868	8.885147006	4.447531342	1.23E-05	0.000120013	2.53463649
KRT80	0.537337387	7.529848957	4.435348919	1.29E-05	0.000125251	2.484169421
NDRG1	0.538945644	9.45110454	4.4317038	1.32E-05	0.000127129	2.469092641
APOC1	-0.579198765	10.26439151	-4.409108597	1.45E-05	0.000138719	2.375877576
CLIC6	-0.512916938	6.919012634	-4.391634193	1.57E-05	0.000148679	2.304074433
SLC5A6	0.533442014	10.239543	4.3891568	1.58E-05	0.000150161	2.293914955
KCNG1	0.501487424	6.726121139	4.379092981	1.65E-05	0.000155912	2.252696215
GBP4	-0.553854633	8.211361496	-4.377257435	1.67E-05	0.000157089	2.245187261
VCAN	-0.507685586	9.182930017	-4.375393342	1.68E-05	0.00015805	2.237564356
SLC7A2	-0.532752324	6.655437177	-4.368769832	1.73E-05	0.000162495	2.210501656
MAL2	0.572745525	9.246712313	4.367971507	1.73E-05	0.000162935	2.207242246
S100A4	-0.512157582	10.98009782	-4.367615302	1.74E-05	0.000163115	2.2057881
CENPW	0.545923558	8.077859487	4.363352495	1.77E-05	0.00016594	2.188393981
SLC7A5	0.575004508	9.753028299	4.328616797	2.05E-05	0.00018921	2.047214011
CDH3	0.678913262	8.823703803	4.320919403	2.12E-05	0.000194424	2.016063134
MB	0.548873658	7.604187728	4.301564959	2.30E-05	0.000209073	1.937952808
PKP1	0.666582041	6.970421985	4.296669191	2.35E-05	0.000213068	1.918243558
DQ893812	0.738971433	9.569455314	4.273059325	2.60E-05	0.000232366	1.823473779
TNFSF13B	-0.51331488	8.679903227	-4.27145006	2.62E-05	0.000233694	1.817030994
PSMB9	-0.503383985	8.484448298	-4.264227148	2.70E-05	0.000240226	1.788140065

ADM	0.646637199	9.201209268	4.207519584	3.42E-05	0.000298021	1.56281938
PDZK1IP1	0.571803303	7.152577011	4.18798079	3.71E-05	0.000320662	1.4858038
KRT16	0.586975569	6.895537057	4.164320741	4.09E-05	0.000350634	1.392969769
CYP1B1	-0.636155256	8.656908721	-4.163638485	4.11E-05	0.00035136	1.390299763
FZD9	0.603140256	7.02983111	4.105749323	5.21E-05	0.000434343	1.165169396
PANX2	0.51196406	6.956090587	4.091837233	5.51E-05	0.000456638	1.111484247
RNASE1	-0.582956745	9.627683017	-4.090770536	5.54E-05	0.000458475	1.1073747
CLDN1	0.769226846	7.975410678	4.085430115	5.66E-05	0.00046762	1.086814621
ARL9	0.521534705	6.963905779	4.085092163	5.67E-05	0.000467947	1.085514346
FBP1	-0.560919332	8.262816454	-4.004864324	7.84E-05	0.000620648	0.779560734
RNF126P1	-0.670229137	6.5183208	-3.978027037	8.73E-05	0.000681505	0.678429536
PHGDH	0.578406155	11.15652603	3.973136028	8.90E-05	0.000692936	0.660064486
BAIAP2L2	0.507511659	6.935455748	3.932648423	0.000104564	0.000801266	0.508819546
CDH1	0.517190634	9.355120659	3.901352415	0.000118295	0.000893002	0.392866695
GBP5	-0.533104724	7.816792877	-3.900736656	0.000118582	0.000894611	0.390593667
CDCA7	0.509346148	8.276027208	3.868793624	0.000134383	0.000995942	0.273122402
SERPINB5	0.737861336	7.693128242	3.856258946	0.000141113	0.001037298	0.227264219
TJP3	0.512707844	7.972006207	3.853484121	0.000142645	0.001047613	0.217130692
IRX3	0.50008069	11.42335741	3.833412522	0.000154207	0.0011187	0.144026698
HEY2	0.587755113	7.329610257	3.823734044	0.000160093	0.001156241	0.108899612
FOXC1	0.821900118	10.58927394	3.790847493	0.000181723	0.001291689	-0.009857605
MMP11	0.704538291	9.884371341	3.776038489	0.00019234	0.001358535	-0.063030814
VTCN1	0.758435766	9.56073591	3.762069089	0.00020289	0.00142192	-0.11301613
SPP1	0.792683533	9.813807555	3.733942522	0.000225809	0.001561266	-0.213147759
KRT18	0.525492382	10.33467473	3.691153084	0.000265411	0.001802547	-0.364167685
PSAT1	0.503005336	8.249664981	3.667734178	0.000289769	0.001948428	-0.446149752
IDO1	-0.528691937	7.249194099	-3.662882983	0.000295072	0.001976988	-0.463072683
SLPI	0.78027219	8.582415617	3.642235768	0.000318677	0.002107332	-0.534869677
EGFR	0.508484655	7.20772774	3.637508812	0.000324325	0.002137723	-0.551254646
COBL	0.500278922	7.995267223	3.521495948	0.000496316	0.003107461	-0.947273846
GABBR2	0.506921979	6.285918199	3.520770375	0.000497622	0.003113233	-0.949713569
HLA-H	-0.500212821	8.858727905	-3.51501618	0.000508086	0.003175442	-0.969045538
KRT81	1.040919541	8.47801224	3.510128862	0.000517136	0.003227035	-0.985442278
ART3	0.785418537	7.356028239	3.479250751	0.000577896	0.003553879	-1.088551538
PRKAR1A	-0.534404302	8.4537319	-3.471001024	0.000595225	0.003647081	-1.115957268
THRSP	-0.516951046	6.322367153	-3.417560238	0.0007198	0.00430643	-1.292034588
HIST1H4H	0.532317975	7.405427327	3.408773085	0.000742479	0.004424731	-1.320745071
MUC16	0.549623895	6.885094376	3.398707889	0.000769275	0.004562105	-1.35354738
HLA-DRB4	-0.590575161	6.855960505	-3.39836572	0.000770201	0.004564268	-1.354660929
KRT23	0.516615847	7.888480894	3.363266154	0.000870982	0.005067834	-1.468336943
VANGL2	0.509929526	7.990347055	3.355753548	0.000894092	0.005177574	-1.492525831
H19	-0.636892736	8.68473299	-3.324531532	0.000996391	0.005692326	-1.592516363
BAMBI	0.553384297	10.11454235	3.32265192	0.001002884	0.005723175	-1.598508277
KANK4	0.577806181	7.912843934	3.30022543	0.001083458	0.006107266	-1.669757778
LOXL4	0.540745271	7.304660849	3.291860321	0.001115024	0.006263463	-1.696219168
BX647938	0.620804152	8.290347865	3.161306319	0.001732417	0.009167621	-2.101092128
ATP6V1B1	0.534335952	8.096551815	3.130042753	0.001921152	0.010021099	-2.195776121
COL11A1	0.541061453	8.825184956	3.085850258	0.002220431	0.011333781	-2.328113302
MSLN	0.839476892	7.745964611	3.039019919	0.002583987	0.012904222	-2.466423454
TFF3	-0.822350557	7.746537992	-3.012765411	0.002810994	0.013864291	-2.543095147
ROPN1B	0.630042264	7.537780376	3.002598598	0.002903713	0.014222649	-2.572617526
GABRP	0.886423073	9.379654908	2.894608118	0.004076862	0.018912043	-2.880393639
ROPN1	0.642413551	7.799106652	2.869747881	0.004401985	0.020189641	-2.949738875
TMSB15A	0.511531632	7.944236385	2.866335813	0.004448406	0.020375706	-2.959212369
KIF1A	0.593581371	7.119778779	2.832888181	0.004927638	0.022211743	-3.05151383
CALML5	0.701263304	8.746923169	2.783892327	0.005714639	0.025045739	-3.184868362
PIP	-0.901863995	7.77654779	-2.739741944	0.0065196	0.027862086	-3.303143177
KRT17	0.591760293	9.246247206	2.634784181	0.008859432	0.036204368	-3.577090631
S100P	0.679932789	9.531622616	2.544876846	0.011436004	0.044730747	-3.803632609
HLA-DRB6	-0.574477836	8.217766362	-2.538769662	0.011633156	0.045349022	-3.818748221
